# Co-assembled perylene/graphene oxide photosensitive heterobilayer for efficient neuromorphics

**DOI:** 10.1038/s41467-022-32725-y

**Published:** 2022-08-25

**Authors:** He-Shan Zhang, Xue-Mei Dong, Zi-Cheng Zhang, Ze-Pu Zhang, Chao-Yi Ban, Zhe Zhou, Cheng Song, Shi-Qi Yan, Qian Xin, Ju-Qing Liu, Yin-Xiang Li, Wei Huang

**Affiliations:** 1 Key Laboratory of Flexible Electronics (KLOFE) and Institute of Advanced Materials (IAM), Nanjing Tech University (NanjingTech), 30 South Puzhu Road, Nanjing, 211816 China; 2 Shandong Technology Center of Nanodevices and Integration, School of Microelectronics, Shandong University, Jinan, 250100 China; 3 State Key Laboratory for Organic Electronics and Information Displays, Nanjing University of Posts and Telecommunications, 9 Wenyuan Road, Nanjing, 210023 China; 4 Frontiers Science Center for Flexible Electronics, Xi’an Institute of Flexible Electronics (IFE) and Xi’an Institute of Biomedical Materials & Engineering, Northwestern Polytechnical University, 127 West Youyi Road, Xi’an, 710072 China

**Keywords:** Two-dimensional materials, Molecular self-assembly

## Abstract

Neuromorphic electronics, which use artificial photosensitive synapses, can emulate biological nervous systems with in-memory sensing and computing abilities. Benefiting from multiple intra/interactions and strong light-matter coupling, two-dimensional heterostructures are promising synaptic materials for photonic synapses. Two primary strategies, including chemical vapor deposition and physical stacking, have been developed for layered heterostructures, but large-scale growth control over wet-chemical synthesis with comprehensive efficiency remains elusive. Here we demonstrate an interfacial coassembly heterobilayer films from perylene and graphene oxide (GO) precursors, which are spontaneously formed at the interface, with uniform bilayer structure of single-crystal perylene and well-stacked GO over centimeters in size. The planar heterostructure device exhibits an ultrahigh specific detectivity of 3.1 × 10^13^ Jones and ultralow energy consumption of 10^−9^ W as well as broadband photoperception from 365 to 1550 nm. Moreover, the device shows outstanding photonic synaptic behaviors with a paired-pulse facilitation (PPF) index of 214% in neuroplasticity, the heterosynapse array has the capability of information reinforcement learning and recognition.

## Abstract (web summary)

Layered heterostructures are promising photosensitive materials for advanced optoelectronics. Here, the authors introduce an interfacial coassembly method to construct large-scale perylene/grahene oxide (GO) heterobilayer for broadband photoreception and efficient neuromorphics.

## Introduction

Layered heterostructures are promising neurotransmitters for artificial optoelectronic synapses due to their ultrasensitive light detection, tunable memory plasticity and ultralow energy potential^1,2^. To achieve high-efficiency synaptic electronics, huge efforts have been made to construct versatile heterostructures and devices, especially low-dimensional (D) heterostructures from 0D–2D and 2D–2D combinations^3^. Among them, 2D heterostructures give more prominence to fascinatingly manipulating photogenerated excitons and charge transport at the interface^4,5^. The coupling of light absorption regions of dissimilar layered matter allows wideband photodetection for the visual perception portion^6,7^, and the unequal bandgaps and confinement transport enable optical memory plasticity for the information processing portion^8,9^. Previous studies show that 2D photonic heterosynapses have a detection range from deep ultraviolet (UV)^10^ to the mid-infrared region^11^, with specific detectivity up to 10^13^ Jones^12^ and energy consumption as low as 10^−9^ W; however, their overall performance is not nearly as good as that of a biological synapse^13,14^.

Conventional layered heterostructure assembly relies mainly on physical stacking and chemical vapor deposition technologies. Several inorganic bilayer heterostructures, including WSe_2_-MoS_2_^15^, WSe_2_-SnS_2_^16^, and GaTe-MoS_2_^17^, have been successfully constructed. However, there are still a few imperfections in the resulting materials, such as grain boundaries with weak homogeneity^18^, unscalable exfoliation with inefficient transfer^19^, and complicated processes with high expense. Furthermore, the relatively high mobility of inorganic layered crystals (100 cm^2^ V^−1^ s^−1^) normally accompanies a relatively high dark current and operating current simultaneously, thus making it hard to enhance the specific detectivity and reduce the power dissipation in man-made synapses, all of which limit the practicality of monolithically large-scale manufacturing and competitive applications. In contrast, carbon-based compounds, especially aromatic-containing organic and graphene oxide (GO), are promising candidates for efficient synapses in neuromorphic electronics, because of tailored chemical structures, tunable photoelectric properties, and facile solution processes^13^. Moreover, these compounds can be assembled into a variety of nanostructures with fascinating features mostly derived from van der Waal (vdW), electrostatic adherence, and supramolecular as well as cooperative interactions^20^. Herein, we report an interfacial coassembly approach to prepare a centimeter-size and uniform photosensitive heterobilayer from perylene and GO precursors, and then utilize the heterobilayer to construct high-performance photo perception and synaptic plasticity with broadband sensitivity, ultralow power, ultrahigh specific detectivity, and high paired-pulse facilitation (PPF) index for information sensing and acquisition.

## Results

### Perylene/GO heterobilayer coassembly and characteristics

**Fig. 1:**
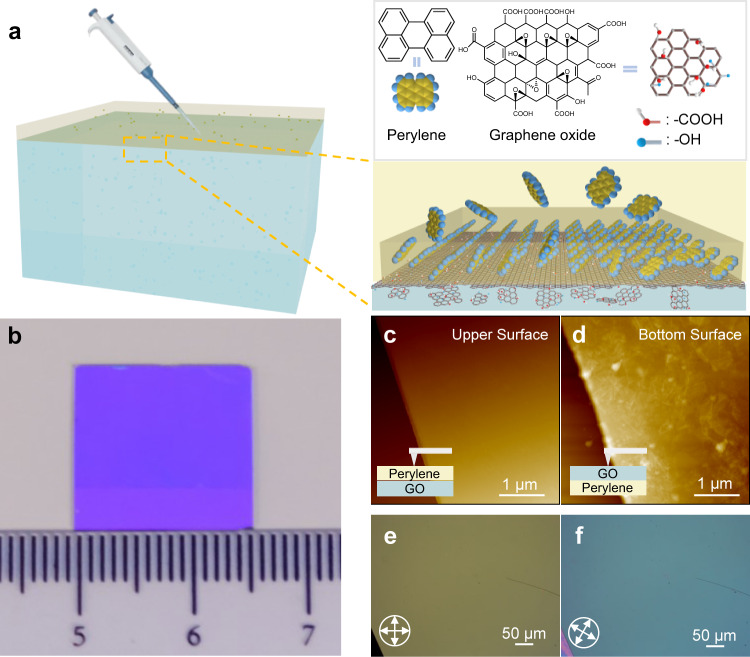
Coassembly and morphology of bilayer heterostructure. **a** Schematic illustration of the coassembly of GO and perylene at the interface. **b** Photograph of the assembled film on a SiO_2_/Si substrate under daylight. AFM images of **c** the upper surface and **d** the bottom surface of the film. **e**, **f** Polarization optical microscope images of the upper layer from the heterostructure.

Figure 1a schematically illustrates the interfacial coassembly of bilayer heterostructure from perylene and GO precursors. Perylene can act as a semiconductor building block to assemble a crystalline layer at the gas-liquid interface with broadband absorption in the visible region^21,22^ due to the relatively strong π–π interactions of aromatic-rich frameworks. GO sheet, as graphene-like aromatic patch, has versatile decoration of polar oxygen-containing hydrophilic groups, such as –OH, =O, and –COOH, which can not only endow GO self-assembly or coassembly with other materials, including aromatic complexes but also enable light overlapping absorption in the visible to UV and near-infrared (NIR) regions for perylene/GO hybridization. According to the step-by-step guideline (Supplementary Fig. 1), the GO solution in methanol solvent was first dropped onto the water surface, and subsequently, the perylene/toluene solution was dropped onto the GO solution. The methanol solution of GO has well dispersion and rapidly spreads at water surface, which is contributed to GO assembly (Supplementary Fig. 2). Meanwhile, the oil/water interface offers a 2D confined space to the heterobilayer assembly (Supplementary Fig. 3).

Under the driving of vdW forces between precursor matter, GO self-assembly and GO-guided perylene assembly simultaneously occur at the interface, resulting in the formation of a continuous centimeter-sized thin film with a homogeneous color distribution over a region of 1.5 cm × 1.5 cm (Fig. 1b and Supplementary Fig. 4b, c), indicating the uniform and scalable growth of such heterostructures. As shown in Supplementary Fig. S4a, only small-size (50 μm) and crackable perylene crystals were achieved under the identical procedure without the addition of GO, suggesting that GO plays a critical role in large-area assembly of perylene. Moreover, by modulating the injected volume of GO solution with a fixed concentration of 2 mg ml^−1^, heterobilayer thickness at the nanoscale level could be precisely controlled from 40 to 400 nm, showing a trend from significant decline to rise due to a progressively increase and saturation evolution of assembled area (Supplementary Figs. 5 and 6). A similar trend for perylene thickness was observed in each bilayer (Supplementary Fig. 7). To verify the generality of our coassembly strategy, other four organic semiconductors, i.e., C8-BTBT, p-MSB, SFX-2-Cz and BMeF, are employed to replace perylene, these resulting crystals also have larger area under the assistance of GO (Supplementary Fig. 8). Therefore, our method paves an universal way for the assembly synthesis of large-area carbon-rich heterostructures.

To preliminarily investigate the morphology and structure of the assembled heterostructure, two films were directly transferred onto 300 nm SiO_2_/Si substrates with front and reverse side contacts. The atomic force microscopy (AFM) images in Fig. 1c, d show that the film has a smooth upper surface and coarse bottom surface that contain discrete nanosheets, with roughnesses of 0.62 nm and 5.58 nm, respectively. Compared with the wrinkled-like surface of the pure GO film (Supplementary Fig. 9), we speculate that the discrete matter on the bottom surface is GO sheets, while the smooth layer should consist of perylene. The GO sheets are stacked into a paper-like membrane that contacts the perylene layer, suggesting layer-by-layer assembly of the heterostructure. Figure 1e, f explains the integrality and crystal characteristics of the perylene layer by using an optical microscope with cross-polarized detection. The color change on the surface is uniform and significant after a 45 degree rotation^23^, revealing its monolithic single-crystal nature.

**Fig. 2:**
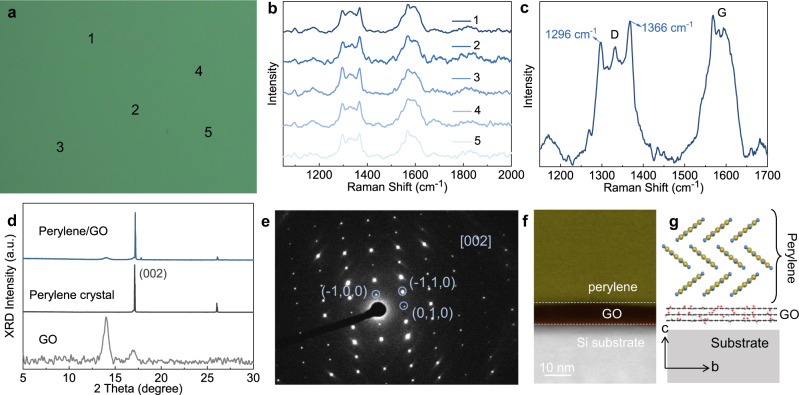
Structural characterization of the perylene/GO heterobilayer. **a** Optical image of the heterostructure film transferred onto a 300-nm SiO_2_/Si wafer. **b** Raman spectra collected from regions labeled 1–5 in **a**. **c** Zoomed-in view of Raman spectra with four-mode absorption. **d** XRD spectra taken from GO, perylene crystals and their combination. **e** SAED image of perylene crystal. **f** Cross-section STEM image of the bilayer perylene/GO structure on a silicon holder. **g** Ordered molecular arrangement of perylene crystal viewed from the *b* axis of the lattice and GO layer; yellow, carbon atom; blue, hydrogen atom.

To further evaluate the film quality and chemical components, the heterostructure was transferred onto a SiO_2_/Si substrate with the GO layer in contact with air. The optical image presents homogeneous contrast over a region of 500 × 375 μm (Fig. 2a), confirming the uniform and scalable growth of such heterostructure. By extracting and comparing five Raman spectra from variable regions in the mapping area, four-mode absorption and negligible variation in each region are summarized (Fig. 2b, c). The two relatively weak peaks of 1296 and 1366 cm^−1^ could be ascribed to the characteristic peaks of the crystalline perylene layer^24^, and the two strong peaks at 1332 and 1583 cm^−1^ are identified as the D-band and G-band of the well-stack GO layer^25,26^, respectively (Supplementary Fig. 10). This result verifies the growth uniformity and chemical constitution of perylene and GO in our large-scale heterostructure. Moreover, the atomic composition in each region was analyzed by energy-dispersive X-ray spectroscopy. As shown in Supplementary Figs. 11 and 12, the bottom layer mainly contains signals of carbon (C) and oxygen (O) elements from GO matter, and the C atomic signals in the upper layer are derived from perylene molecules.

The detailed crystal structure of the obtained heterostructure was symmetrically assessed by X-ray powder diffraction (XRD), selected area electron diffraction (SAED) and cross-sectional scanning transmission electron microscopy (STEM). In XRD, the diffraction peak at 2*θ* = 17° is assigned to the (002) diffraction pattern for perylene (Supplementary Fig. 13), manifesting the *ab* plane of the crystal film parallel to the substrate. All characteristic peaks of the perylene crystal and GO layer (2*θ* = 14° and 17°) appear without any unidentified peaks (Fig. 2d), suggesting that no new phase formed in the coassembly process. This conclusion can be further confirmed by the SAED of perylene crystals exfoliated from the heterostructure (Fig. 2e). The remarkable bright spots in SAED, with the largest crystal face of (002), match well with the above XRD result of perylene. From the cross-section image of the bilayer heterostructure measured by STEM, the thickness of GO layer is ∼3 nm (Fig. 2f), which is in consistent with the AFM results in Supplementary Fig. 7.

Based on the above analysis, the inner molecular packing of heterostructure is presented in Fig. 2g and Supplementary Fig. 14. The crystalline perylene adopts herringbone-like packing, with a strong π–π partial overlap of 3.481 Å between adjacent molecules in the *ab* plane, i.e., (002) crystal face, resulting in parallel stacking to the surface of GO layer. In this stacking, the molecular long axis of perylene was tilted by 40.5° with respect to the crystallographic *c*-axis, accompanying strong π–π interaction between perylene and GO. In particular, multiple hydrogen bond interactions, such as C=O…H and O-H…H, may also exist between multiple oxygen-containing groups of GO and the edge face of perylene. The abundant π–π interactions and hydrogen bond interactions can form a 2D network of intermolecular forces, which reduce the nucleation energy of precursors for efficient 2D assembly and also afford strong cohesion between the two layers within the heterostructure. Such advances in combining two matters and abundant interactions endow the hybrid with strong light-matter coupling and ultrabroadband light absorption from UV to visible/NIR regions compared to each single-component (Supplementary Fig. 15), which are suitable for high-performance optoelectronics.

### Photosensitive characteristics

**Fig. 3:**
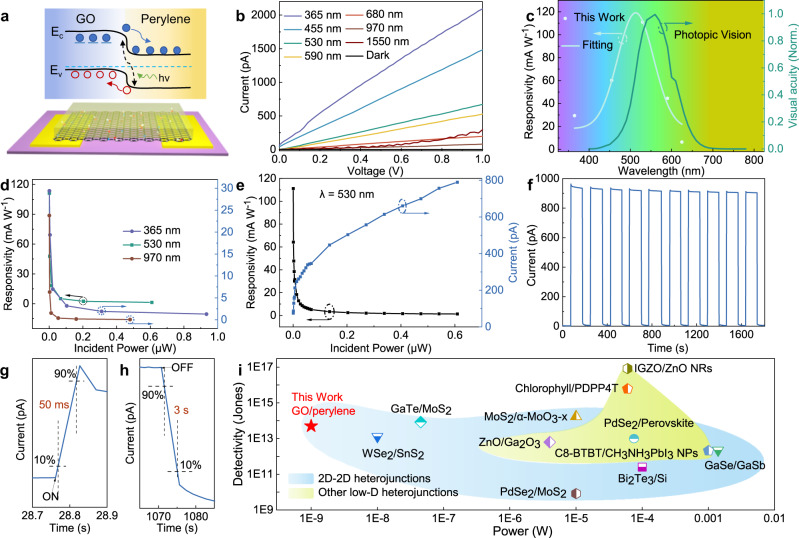
Perylene/GO heterobilayer photodetector. **a** Structural scheme and energy band diagram of the in-plane heterostructure device. **b** Photoresponse behaviors under UV, visible and NIR light stimuli. **c** Visual sensitivity of the human eye and visible light responsiveness of the detector. **d** Photoresponsivity as a function of incident light power at 365, 530, and 970 nm. **e** Dependence of photocurrent and photoresponsivity on incident power at 530 nm. **f** Time-resolved photocurrent of the detector excited at 365 nm. **g**, **h** The rise and fall relaxation time of the photocurrent. **i** Comparison of specific detection rate and power consumption of heterostructure photodetectors in the previous literature.

As discussed above, we have achieved a high-quality large-area perylene/GO heterobilayer. To explore the physical properties, we have assembled a 40 nm thick transparent heterostructure film (Supplementary Fig. 16) and studied its photoelectric effect. Figure 3a and Supplementary Fig. 17 show the configuration of the in-plane heterostructure device and its corresponding band diagram. Since graphene has atomically electroactive surface, its Fermi level can be tuned by heteroatom doping or environmental change, making it promising for versatile detection^27^. Oxygen decoration as an electron acceptor normally leads to p-type doping and induces abundant defects in GO, with an average energy level of 4.9 eV measured by an UV photoelectron spectrometer (Supplementary Fig. 18), which provides hole transport pathways for carbon electronics. The Fermi and HOMO levels of perylene are calculated at 4.9 and 6.1 eV, respectively, a Type II band structure can be formed due to the energy level mismatches in heterostructure. Under illumination, the heterostructure absorbs light energy to generate electron-hole pairs, and these photo excited electrons at the interface are trapped within defect sites, while holes transport along the pathways, thus producing a photoresponse current^12,28–30^.

The heterostructure device exhibits wide-band photoperception abilities (365–1550 nm, Fig. 3b and Supplementary Fig. 19) due to the synergic light absorption of perylene and GO. The photoresponsivity (*R*, defined by *I*_*ph*_*/P*) as a function of wavelength is depicted to examine the photoelectric conversion performance (Fig. 3c and Supplementary Fig. 20), where *I*_*ph*_ is the photocurrent and *P* is the power of incident light. Interestingly, the detector has a normal distribution of photosensitivity within the visual range, with the highest photoelectric conversion efficiency of ∼500 nm. This trend is analogous to the retina-like photoperception function for biomimetic eyes^31,32^.

To gain more insight into the photodetector performance, the impact of incident light power on the photoresponsivity at different excitation wavelengths was studied. In the visible region at 530 nm, the device exhibits the highest responsivity of 110 mA W^−1^ at an excitation power of 0.01 μW, indicating its outstanding ability for weak light detection. However, the responsivities in the UV and NIR regions are relatively low, with values of 30 and 24 mA W^−1^, respectively (Fig. 3d). Figure 3e illustrates the power-dependent photoresponse under 530 nm light illumination. The photocurrent has a positive nonlinear relation with the power intensity, while the corresponding responsivity decreases exponentially. With the increase of power, more photoexcited carriers will generate and contribute to a higher electric field with an opposite built-in field in the junction, which can hinder the separation of more electron-hole pairs, thus present a smaller responsivity^28,30,33^. Importantly, the photodetector shows excellent repeatability and stability of the photoelectric switch (Fig. 3f). Moreover, the maximum photocurrent changed slightly with the thickness of heterostructures, which facilitate the homogeneity of device production (Supplementary Fig. 21). Based on the rising and falling steps of the time-resolved photo response curves, the rise time (*τ*_r_) and fall time (*τ*_f_), defined as the time required for photocurrent rise/drop between 10 and 90%, are 50 ms and 3 s, respectively (Fig. 3g, h). The slow τ_f_ is probably attributed to the decay of persistent photocurrent induced by excess electrons trapped in GO defects, which makes the heterostructure a promising synapse for neuromorphic electronics.

As a figure of merit, the specific detectivity D* is widely used to assess the detection capability of photodetectors. In photodiodes, the generation-recombination noise can be ignored because the carriers run out in the space charge region, and the shot noise in the current passing through the junction is the intrinsic noise^34^. The detectivity of our photodetector can be calculated using the following equation:

1 $${D}^{*}=\frac{\sqrt{A}{R}_{i}}{\sqrt{2e{I}_{{{{{{{\rm{dark}}}}}}}}}}$$

where *I*_dark_ is the dark current, *R*_*i*_ is the photoresponsivity, and *A* is the effective area of detector (7.2 × 10^−4^ cm^2^). Due to the relatively low conductivity of GO matter^35^, the dark current of our device is as low as 0.03 pA (Fig. 3b and Supplementary Fig. 22). Together with its high photoresponsivity (110 mA W^−1^) at 530 nm, the detector has an ultrahigh D* of 3.1 × 10^13^ Jones. Moreover, the photodetector shows an ultralow operating power of 10^−9^ W. Figure 3i summarizes the D* and power consumption *P* of emerging photodetectors based on GO/perylene and some previously reported heterostructures. Impressively, our GO/perylene detector possesses the outstanding D* and lowest *P* among all these devices (Supplementary Table 1). It is concluded that emerging 2D heterostructures are more competitive than most traditional heterojunctions in the biomimetic retina for light detection.

### Photonic synaptic characteristics

The obtained detector has wideband light perception, slows signal decay, ultrahigh specific detectivity, and ultralow energy consumption, which endow the device to emulate visual perception and synaptic plasticity, such as biological neurons, with the characteristics of an excitatory postsynaptic current (EPSC), spike-intensity dependent plasticity (SIDP), spike-number dependent plasticity (SNDP), PPF, short-term memory, and long-term memory^36,37^. With a single UV pulse (0.144 μW mm^−2^, 15 s) under 1 V, a large number of photogenerated carriers are generated and transferred from perylene to GO, resulting in a surge of EPSC (Supplementary Fig. 23). After switching off the light, the photocurrent declines with a long-term relaxation process due to the slow release of trapped carriers in defects. Supplementary Fig. 24 demonstrates the EPSC of two successive weak light pulses (0.144 μW mm^−2^, Δ*t* = 2 s). The current peak from the second pulse (*A*_2_) is higher than that from the first pulse (*A*_1_), and the ratio *A*_2_*/A*_1_ is called PPF^38^. In biology, PPF is a critical short-term plasticity enhancement process and is essential for the temporal decoding of visual signals^39,40^. In neurology, STP is usually of the same order of magnitude as LTP, it should have a significant effect on local neural computing^41^.

**Fig. 4:**
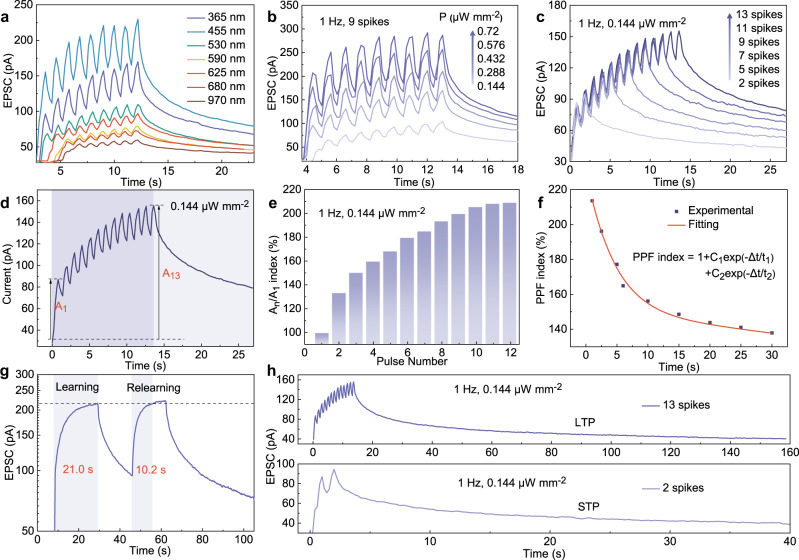
Phototunable synaptic properties. EPSCs in response to nine successive light pulses **a** under UV, visible and infrared light sources and **b** under different powers with a fixed wavelength at 365 nm and a frequency of 1 Hz. **c** Influence of the pulse number on EPSCs under fixed power at 365 nm (0.144 μW mm^−2^_,_ 1 Hz). **d** Measured photocurrent generated by multiple light pulses and the definition of the index (*A*_13_/*A*_1_). **e** The evolution of the *A*_n_/*A*_1_ index with the pulse number. **f** PPF index plotted as a function of interspike interval. **g** The learning-experience behavior of the photonic synapse device. **h** STP and LTP processes under 2 and 13 pulses.

Notably, the artificial heterosynapse shows evident PPF behavior during successfully pulsed light with ultrabroadband wavelengths from UV to NIR (Fig. 4a). Both SIDP and SNDP behaviors were investigated in Fig. 4b, c. The overall EPSC is proportional to light intensity and pulse number, and the refresh signal after receiving the latter pulse is higher than that after receiving the former pulse, indicating synaptic plasticity enhancement by raising learning intensity and times. This enhancement is probably attributed to the superposition of subsequent excited carriers with partial carriers left before the following pulse, because of efficient generation and slow decay of carriers under short pulse interval^42,43^. To quantify the PPF, the amplitudes of the postsynaptic current under multiple pulses are calculated in Fig. 4d, e, where the *A*_n_/*A*_1_ index is denoted as the ratio of the first and last spike currents. With the increase in pulse number, the growth rates of amplitude amplification slow until approaching a saturation state (209.9%). Figure 4f explores spike-time-interval-dependent plasticity with variable intervals ranging from 0.1 to 30.0 s. The index progressively declines with increasing interval, and this attenuation curve matches well with the double exponential function, showing a great ability to mimic biological PPF behavior^38^. The photonic synapse has a maximum PPF up to 214%, the value exceeds the best performance of synapses from 2D heterostructures (Supplementary Table 2). A larger PPF index can lead to a higher temporal resolution, making the constructed artificial neural network (ANN) model more efficient and accurate in processing temporal information^44^.

According to Ebbinghaus’s theory^45^, relearning information in biological synapses takes less time than initial learning. To emulate this learning-experience behavior, the device was irradiated by two adjacent 365 nm light pulses with identical intensities (0.432 μW mm^−2^) and tunable intervals. As shown in Fig. 4g, it takes 21.0 s to reach the 214 pA level during the original learning, and then the recorded signal decays exponentially. In contrast, subsequent learning only requires 10.2 s to approach the same cognitive level, which is similar to human-like learning^46^. Furthermore, the transition from the STP to LTP process is described in Fig. 4h. By applying two successive pulses with a pulse frequency of 1 Hz, a pulse width of 0.5 s, and a pulse intensity of 0.144 μW mm^−2^_,_ the memory strength of the signal declines rapidly to 40 pA in a short interval of 30 s, which is defined as the STP process. When 13 successive pulses are applied, the signal descending to the reference level requires a longer decay time of 160 s, resulting in the formation of LTP. Therefore, the STP can be progressively converted to LTP by modulating the number of pulse learning^47^.

**Fig. 5:**
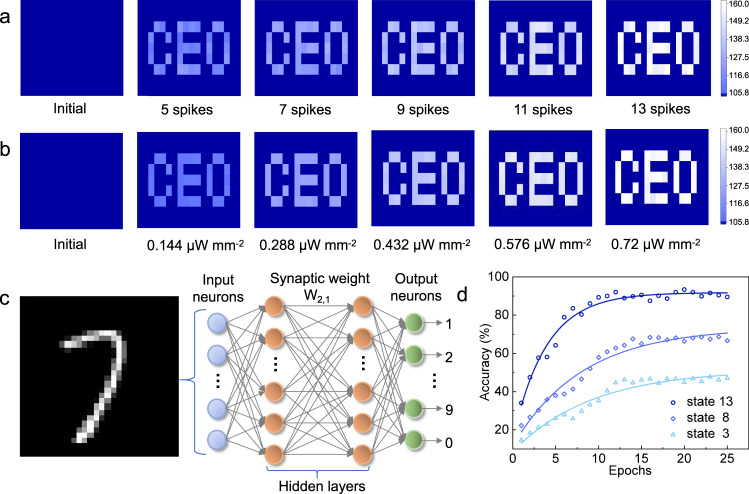
Image memorization and recognition. Measured weights of a “CEO” pattern image in the initial state and **a** after training with 5, 7, 9, 11, and 13 spikes under 365 nm light with a power density of 0.288 μW mm^−2^ (pulse width, 500 ms; pulse interval, 500 ms), **b** after training with various power densities of 0.144, 0.288, 0.432, 0.576, and 0.72 μW mm^−2^ (pulse width, 500 ms; pulse interval, 500 ms) under nine pulses at 365 nm. **c** Schematic illustration of a light-derived neural network. **d** Accuracy calculation of the network under different pulse states.

The outstanding learnability enables artificial synapses to mimic visual learning for high-quality image sensing and deep learning. To this end, a random pattern image “CEO” as signal input consists of 10 × 10 synapse pixels, and the gray of each pixel represents the measured ESPC value. Figure 5a illustrates the evolution of pixel gray over pulse times in the mapping area simulated by synapse arrays. After applying the training of 0, 5, 7, 9, 11 and 13 pulse spikes with a weak (0.288 μW mm^−2^) light, the weighted graphs of learning patterns have enhanced sharpness and likeness to these input letters. As the pulse number reaches a certain level, the distinct target image becomes uniform and easily recognizable. Furthermore, to assess the memory effect under variable learning intensity, we investigated the influence of illumination intensity on pixel gray (current) under nine fixed pulses at 365 nm light. As the intensity of learning increases to 0.72 μW mm^−2^, a clear shape of the “CEO” figure was also achieved (Fig. 5b), meaning that the efficiency of visual learning can be accelerated by enhancing light intensity. These features are analogous to the image learning of biological neurons.

To assess the reliability of our photonic synapse in image cognition, the accuracy of an ANN is simulated and calculated by utilizing the heterosynapse as a neuron. In biological neural networks, both LTP and short-term depression (LTD) processes are required to depolarize and reverse neurons, respectively, which can dynamically balance the synaptic weight for efficient signal processing^48,49^. The constructed heterosynapse not only has an LTP feature but also possesses LTD behavior under the unipolar pulsed bias (Supplementary Fig. 25). Their corresponding nonlinearities as learning efficiency and recognition rates are extracted by fitting the experimental data, with a maximum value of 1.98 for LTP and 4.21 for LTD (Supplementary Fig. 26). This bidirectional modulated function with appropriate nonlinearity allows them to construct an efficient ANN^50^.

Figure 5c depicts the schematic light-derived ANN architecture comprising 784 inputs, 2 hidden and 10 outputs. A 28 × 28 handwritten pattern recognition was adopted from the database of the Modified National Institute of Standards and Technology^51^, with one photonic synapse at each intersection of the crossbar structure (Supplementary Fig. 27). According to the above model, the accuracy of recognition was calculated by using 60,000 training and 10,000 test images as well as 64 batch sizes at each epoch. In contrast to unidirectional modulation, the bidirectional strategy shows a higher accuracy of 85%, indicating advanced cognitive capability for more complex neuromorphic information (Supplementary Fig. 28). Figure 5d demonstrates the high dependence of cognitive accuracy on epochs under different pulse times. The overall accuracy for all pulse states improves at a faster rate during the initial epoch, and then tends to saturate at ~15 epochs. As the number of pulses increases from 3 to 8 and 13 times, the responding saturated accuracy increases from 50 to 60% and 80%, respectively. The results were clarified by simulating number “7” along with the evolution of learning stages (Supplementary Fig. 29).

## Discussion

In summary, we demonstrated a facile interfacial coassembly of a large-area photosensitive heterobilayer and utilized it as an optoelectronic converter for neuromorphic electronics. The heterobilayer was assembled from GO and perylene at the liquid–liquid interface, with the merits of centimeter-scale, high uniformity and broadband absorption from visible to NIR regions. These characters ensure the exceptional photoperception of planar heterostructure devices, with an ultrahigh detectivity of 3.1 × 10^13^ Jones, ultralow power consumption of 10^−9^ W, and typical photonic synaptic behaviors, including EPSC, SIDP, SNDP, PPF, STP, LTP, and LTD under appropriate light and electrical pulses, which can serve as bioinspired photoreceptors and synapses synchronously. Specifically, this heterosynapse as an artificial neuron can be implemented into a neuromorphic network that is capable of visual learning and recognition, promising efficient temporal information decoding owing to the high PPF feature. Our work provides an effective and universal strategy for wafer-scale production of economical low-dimensional heterostructures for bioinspired optoelectronics.

## Methods

### Materials

Perylene, Toluene, Methanol were purchased from Aladdin. Ultrapure water (18.2 MΩ cm) was prepared by laboratory TANKPRO ultrapure water instrument. The above raw materials are used directly without further purification.

### Preparation of GO, perylene crystal and perylene/GO heterobilayer

GO in this work were synthesized via the modified Hummer method. To prepare the 2 mg ml^−1^ GO/methanol solution, the synthetic GO was dissolved in methanol solvent, fully dissolved, ultrasonic, centrifuge. To prepare the perylene crystals, 150 µl 1 mg ml^−1^ perylene/toluene solution was dropped on the water surface (15 ml) in a weighing bottle (50 × 35 mm). To assembly the perylene/GO heterobilayer, 150 µl 1 mg ml^−1^ perylene/toluene solution was dropped on the water surface (15 ml) with 50 µl 2 mg ml^−1^ GO/methanol solution in a weighing bottle (50 × 35 mm). Then the weighing bottles was placed in a reaction tank and maintained at room temperature, enabling the slow evaporation of toluene. After the full evaporation of the organic solvent, large-area perylene/GO heterostructures were formed on the water surface.

### Heterobilayer characterizations

POM (NIKON, LV100ND), SEM (JSM-7800F) and AFM (Park, XE-70) were used to characterize the morphologies and structures of perylene/GO heterostructure. UV–vis absorption spectra were recorded on a UV-1780 spectrophotometer. The growth of perylene on GO were confirmed by Raman spectroscopy measurement using a Renishaw 2000 CCD spectrometer coupled to an Olympus BH-2 confocal microscope, and using the 633 nm line of a He-Ne laser as the excitation source to avoid absorption and resonance effects. The powder XRD patterns were performed on a Rigaku Smartlab X-ray diffractometer. The operating 2*θ* angle ranges from 5 to 30 Å, with the step length of 0.02 Å. The transmission electron microscopy and SAED studies were performed in a USA FEI TF20, operating at an accelerating voltage of 200 kV.

### Heterobilayer transfer and device fabrication

The assembled heterobilayer could be readily transferred from the water surface to any target substrates. In total, 80 nm Au was steamed onto a washed 300 nm SiO_2_/Si substrate to form an electrode with a channel of 50 μm with the help of a custom-made mask. The target substrate (300 nm SiO_2_/Si wafer with Au electrodes) was inserted obliquely into the water solution near the bottom of the heterostructure, followed by lifting the heterostructure from the solution surface. Then, the sample was put on a hot plate and dried at 50 °C.

### Photoresponse and photosynapse measurements

All electrical and optical measurements were made through Keithley 4200 SCS semiconductor parameter analyzer under ambient conditions, the detailed stepwise of device testing is displayed in Supplementary Fig. 30. Light pulses with different irradiation intensities, frequencies and wavelengths come from high-power LED drivers (THORLABS, DC2200 Terminal) and a series of LED sources covering UV, visible, and NIR (THORLABS, M365L3, M455L4, M530L4, M590L4, M625L4, M680L4, M970L4, M1050L2, M1300L3, M1550L3). The light source system was mounted in the Keithley 4200 SCS shielding box to avoid interference from external light signals.

The optoelectronic characteristics of the device were measured through Keithley 4200 SCS: to measure the photocurrent generated by the device, different bias voltages were applied through the probe to the two terminals of the device. The effects of different modes of light pulse on photocurrent were investigated by time-current mode. By adjusting the irradiation intensity of light source, photodetector and optical synapse performance can be both explored. The optical response of artificial vision arrays was studied by studying the synaptic response of each synaptic device to different optical signals. Multiple control groups were established in the tests by adjusting the illumination conditions, including the duration of light, pulse frequency, radiation intensity and the number of pulses.

### Weight updating method

The hand-written digital graph (28 × 28 pixels) in the MINST dataset was converted into 784-dimensional vector (x_0_–x_783_) and entered into 784 input layer neurons. The current neurons were converted to the output neurons (γ_0_–γ_9_ corresponds to the output value of the numbers 0–9 respectively) by the SIGMOD function and the weight and bias of each edge. The calculation rules were expressed as:

2 $$\gamma=\sigma \left(\sum {x}_{i,j}{W}_{j,k}+b\right)$$

Here, *x*_*i, j*_ was the vector value of the *j*th neuron at layer *i*, *W*_*j, k*_ was the weight connecting *x*_i, j_ to the neuron at the next layer, and *b* was the bias value. Sigmoid was chosen as the activation function $\sigma ()$. After the calculation, the weight was updated by the difference between the realistic output value *γ*_*r*_ and the expected output value *γ*_*e*_. Here, the weight value *W* was represented by the different conductive states *G*_*n*_ of the photosynapse device. The calculation method of Δ*W* was based on the characteristic parameters of the photosynapse device. The calculation is based on the following formula:

3 $${G}_{n+1}={G}_{n}+\bigtriangleup G={G}_{n}+{\alpha }_{p}{e}^{-\beta \frac{{G}_{n}-{G}_{{\min }}}{{G}_{{\max }}-{G}_{n}\,}}\,\left({{{{{\rm{Potentiation}}}}}}\right)$$

4 $${G}_{n+1}={G}_{n}+\bigtriangleup G={G}_{n}-{\alpha }_{d}{e}^{-\beta \frac{{G}_{n}-{G}_{{\min }}}{{G}_{{\max }}-{G}_{n}}}\;({{{{{\rm{Depression}}}}}})$$

Here, *G*_*n*_ represented the conductivity of synaptic devices in the nth states, *G*_*n+*1_ represented conductivity after update. Further, parameters *α* and *β* represented the step size of the conductance change and the nonlinearity (NL) value, respectively. The NL values for the potentiation and depression were calculated by fitting LTP/LTD curves in Fig. S26. The mini-batch gradient descent algorithm was used to update the weight by backpropagation. The above simulation process was realized by code written in Pyhton language.

## Supplementary information

Supplementary Information

## Data Availability

The data that support the findings of this study are available from the corresponding authors upon reasonable request.
