# Supplementary material for: Co-assembled perylene/graphene oxide photosensitive heterobilayer for efficient neuromorphics
Source: Nat Commun. 2022 Aug 25;13:4996. doi: 10.1038/s41467-022-32725-y (PMC9411554; doi:10.1038/s41467-022-32725-y)
Supplement: Supplementary file 1 — Supplementary Information [file 41467_2022_32725_MOESM1_ESM.pdf]

Supplementary Information

Co-assembled Perylene/Graphene Oxide Photosensitive Heterobilayer for Efficient Neuromorphics

He-Shan Zhang[1,#], Xue-Mei Dong[1,#], Zi-Cheng Zhang[1], Ze-Pu Zhang[1], Chao-Yi Ban[1], Zhe Zhou[1], Cheng Song[1], Shi-Qi Yan[2], Qian Xin[2], Ju-Qing Liu[1*], Yin-Xiang Li[1*], Wei Huang[1,3,4*]

[1]Key Laboratory of Flexible Electronics (KLOFE) & Institute of Advanced Materials (IAM), Jiangsu National Synergetic Innovation Center for Advanced Materials (SICAM), Nanjing Tech University (NanjingTech), 30 South Puzhu Road, Nanjing 211816, China.

[2]Shandong Technology Center of Nanodevices and Integration, School of Microelectronics, Shandong University, Jinan 250100, China.

[3]State Key Laboratory for Organic Electronics and Information Displays & Institute of Advanced Materials (IAM), Nanjing University of Posts & Telecommunications, 9 Wenyuan Road, Nanjing 210023, China.

[4]Frontiers Science Center for Flexible Electronics, Xi'an Institute of Flexible Electronics (IFE) and Xi'an Institute of Biomedical Materials & Engineering, Northwestern Polytechnical University, 127 West Youyi Road, Xi'an 710072, China.

[#]These authors contributed equally to this work.

Corresponding Author: Email: iamjqliu@njtech.edu.cn; iamyxli@njtech.edu.cn; vc@nwpu.edu.cn

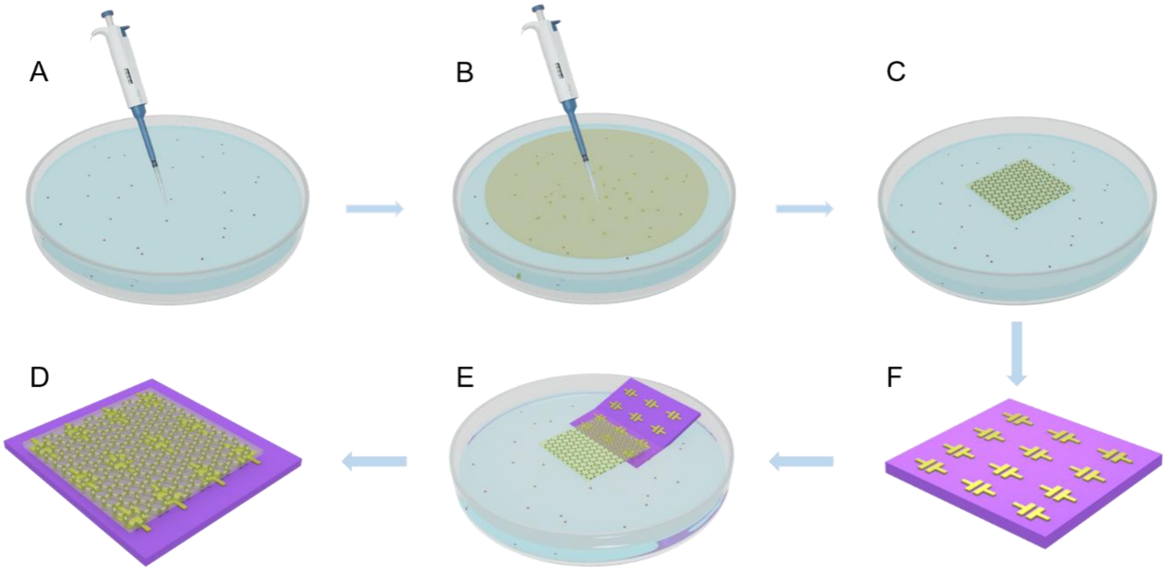

Supplementary Figure 1. The schematic diagrams of growing perylene/GO heterostructure and corresponding device. A) Dropping GO solution onto water surface, B) Dropping perylene solution onto water surface with GO solution, C) large self-assembled heterostructure on water surface, D) Steam Au electrode deposition on SiO_2/Si substrate, E) Transfer the perylene/GO heterostructure to the substrate with Au, F) Device diagram of perylene/GO heterostructure.

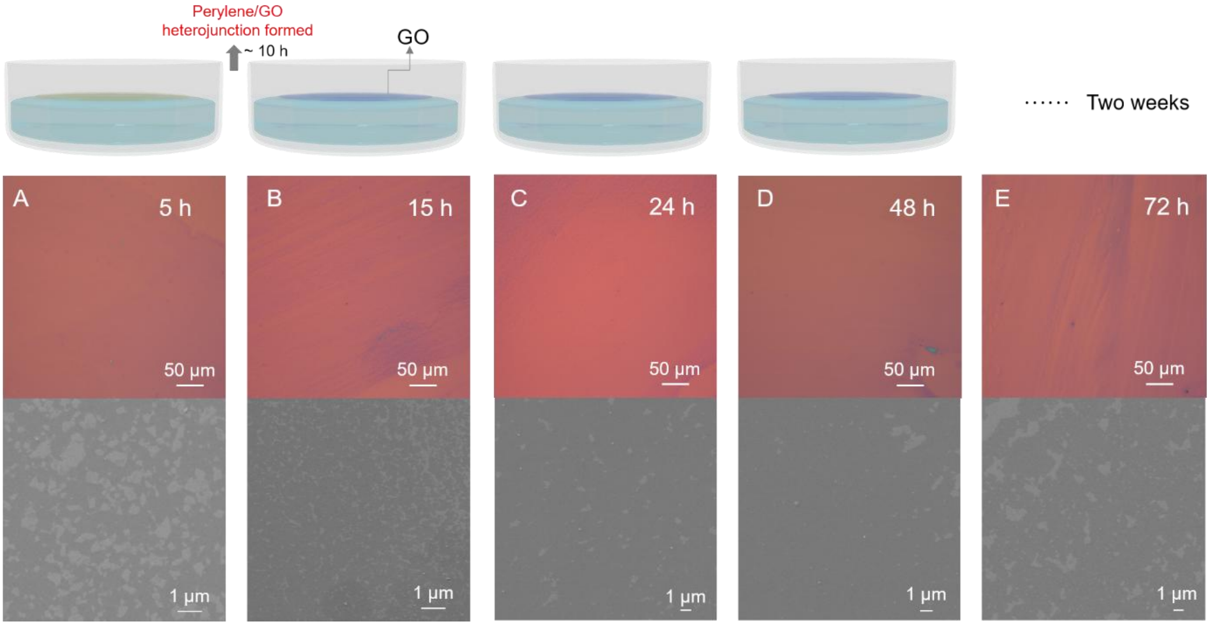

Supplementary Figure 2. The time-dependence contrast experiments of GO morphology with the addition of toluene. It is found that the GO film was remain stable after 72 h (3 days). Upon the quiescent time exceeds 2 weeks, a small amount of GO begins to sinks into the sub-phase. Actually, in our work, the co-assembly of GO and perylene crystal could be completed and transfered within 10 hours.

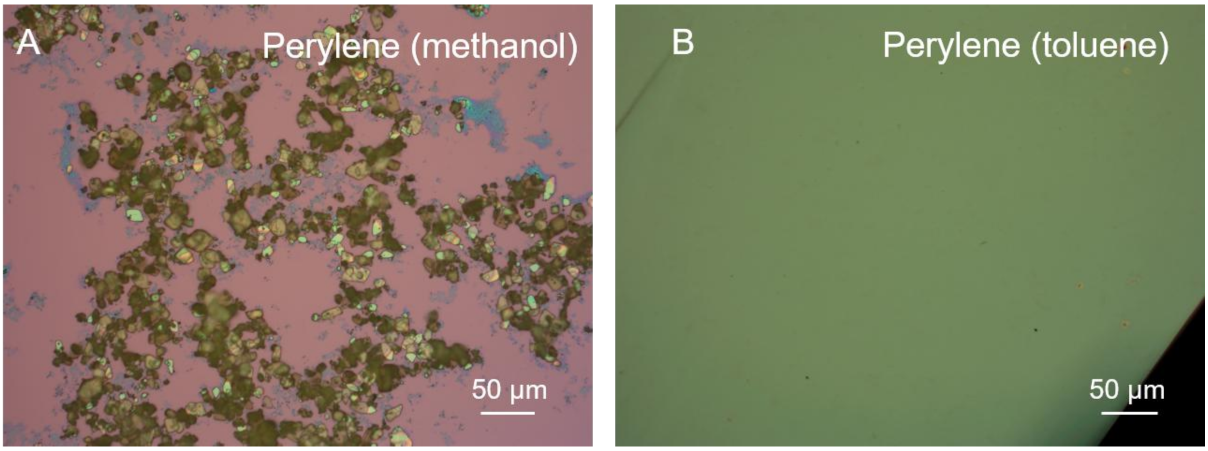

Supplementary Figure 3. Perylene crystal obtained in A) methanol and B) toluene solvent. During crystal growth and even before nucleation, the low boiling point of methanol solvent leads to complete volatilization, which prevents further crystal growth (Supplementary Figure 3A). The low evaporation rate due to the high boiling point of toluene solvent ensured sufficient time for self-assembly of perylene molecules to obtain two-dimensional perylene crystals with high quality and large area (Supplementary Figure 3B).

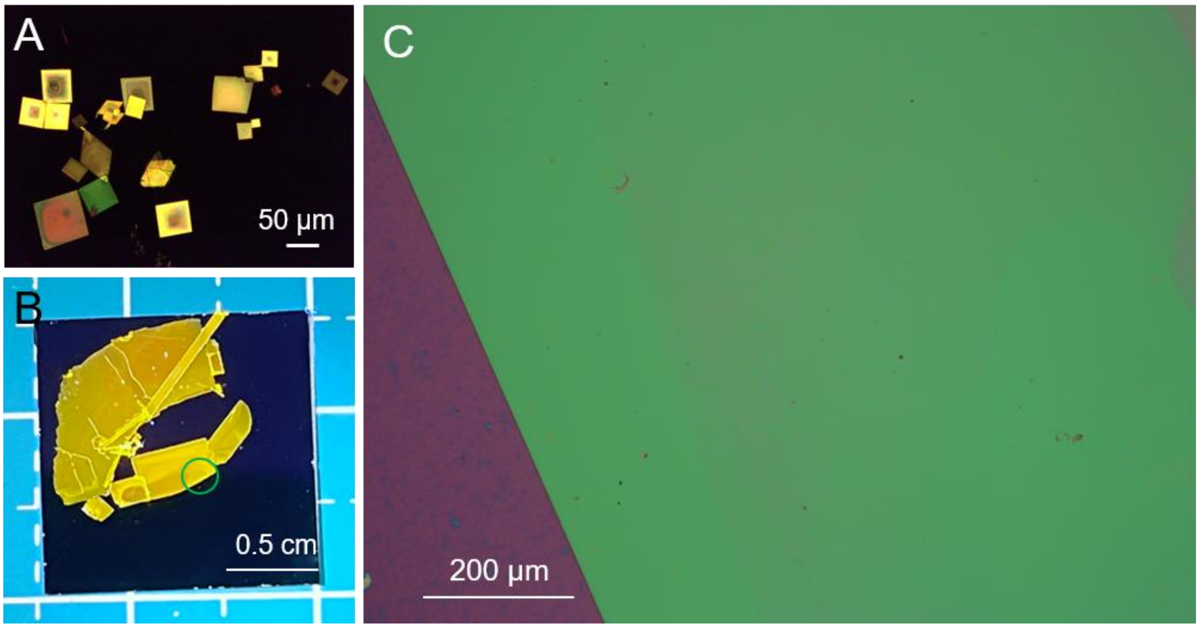

Supplementary Figure 4. **Crystal growth contrast experiment.** Perylene crystal prepared A) on pure water surface and B) on GO solution surface. C) magnified optical microscope images of perylene crystal in B). With the addition of GO solution, larger 2D perylene crystals could be obtained. On the one hand, GO nanosheets provided site induction to promote the growth of molecular crystals, and on the other hand, electrostatic adsorption and hydrogen bonding between oxygen-containing functional groups and organic molecules further accelerated the growth.

Serial number	A	B	C	D	E	F
Perylene (mg mL^{-1})	1	1	1	1	1	1
GO (µL)	5	20	40	50	300	500
Heterojunction thickness (nm)	396	280	105	40	93	162

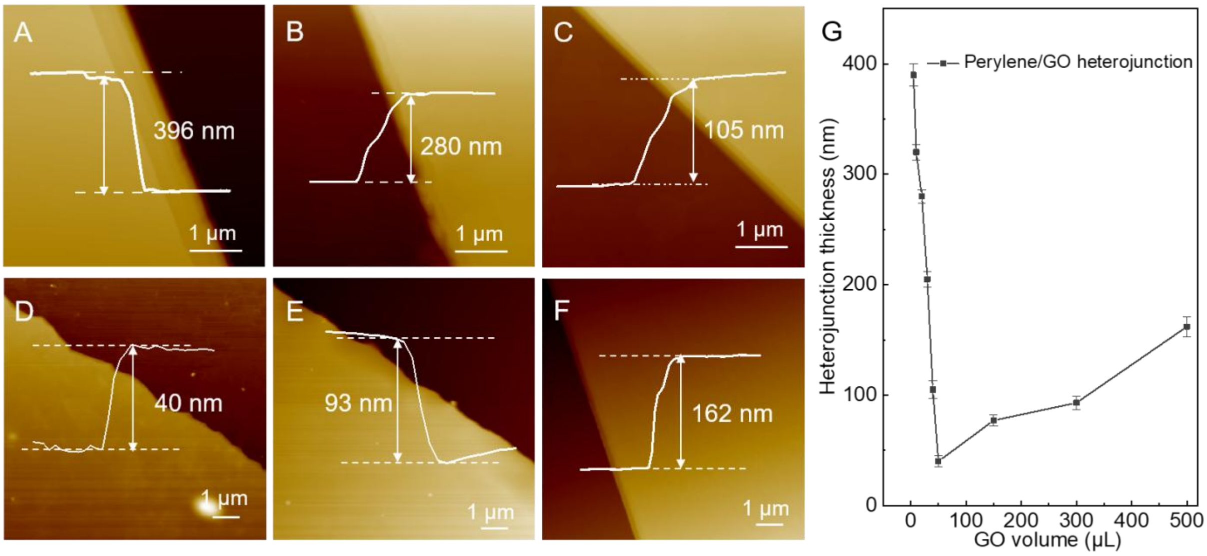

Supplementary Figure 5. **Regulation of heterostructure thickness.** AFM images of perylene/GO heterostructure with the different thicknesses. By co-self-assembly with 5, 20, 40, 50, 300, 500 µL GO solution, A) 396, B) 280, C) 105, D) 40, E) 93, F) 162 nm thick perylene/GO heterostructure was formed and G) thickness data statistics.

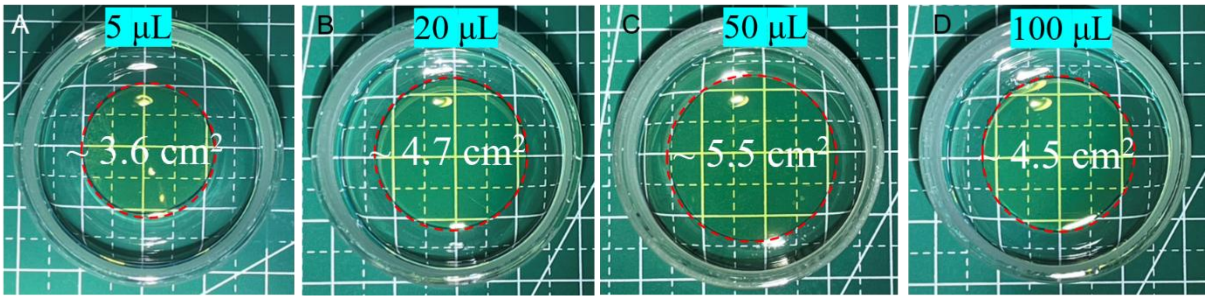

Supplementary Figure 6. A–D) Photographs of the spreading of perylene/toluene solvent on the GO solution surface with different volume of GO.

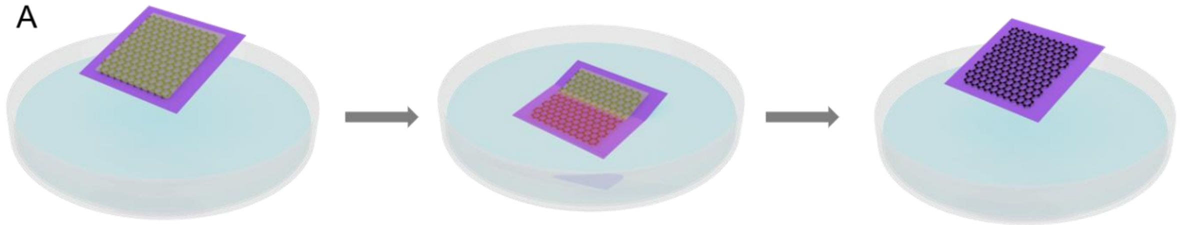

Serial number	B	C	D	E
Perylene (mg mL^{-1})	1	1	1	1
GO (μL)	5	40	50	300
Heterojunction thickness (nm)	396	105	40	93
GO thickness (nm)	2	3.5	4.1	4.5
Perylene thickness (nm)	394	101.5	35..9	88.5

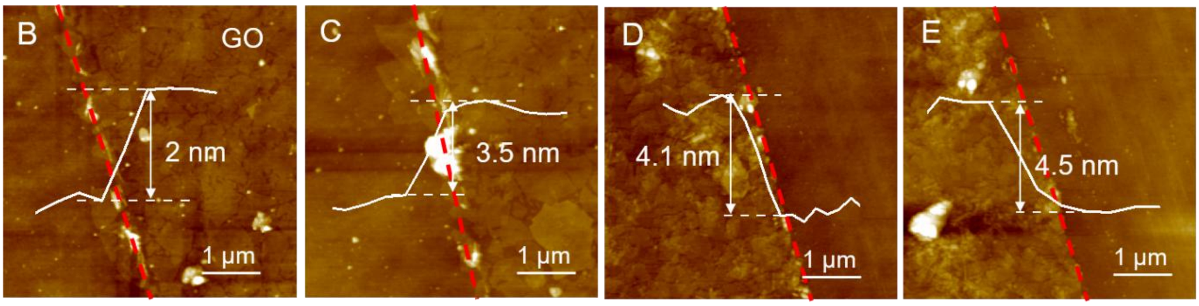

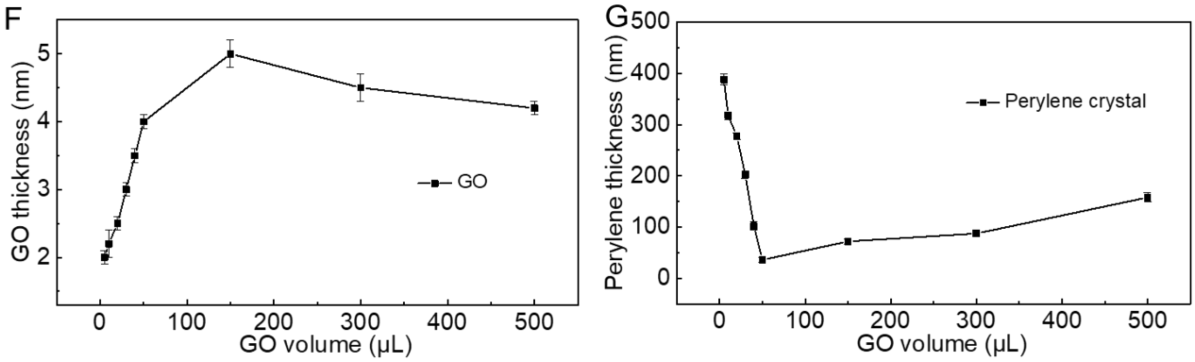

Supplementary Figure 7. Thickness statistics of the separate layers in the bilayer heterostructure. A) The schematic diagram of removing perylene crystals from heterojunction by toluene solution. B-E) The AFM images of GO with 2, 3.5, 4.1 and 4.5 nm thickness. F-G) The thickness statistics of GO and perylene crystal with an error bar, respectively.

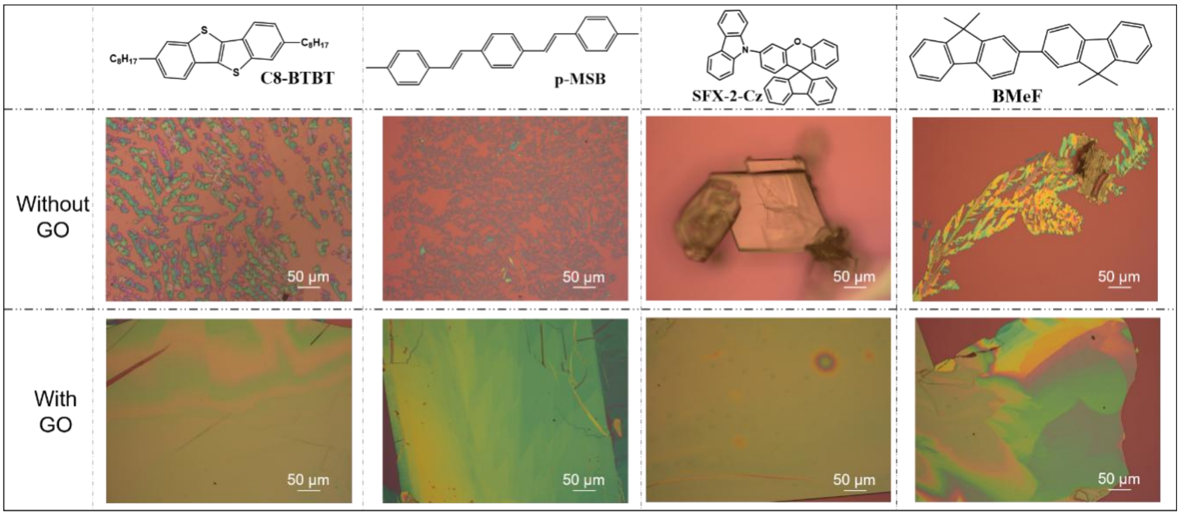

Supplementary Figure 8. **Generality of GO-assisted interfacial co-assembly strategy.** A) Chemical structures of the four organic semiconductors of C8-BTBT, p-MSB, SFX-2-Cz and BMeF. OM images of the fabricated crystals co-assemblied under the condition (B) without GO, and (C) with GO.

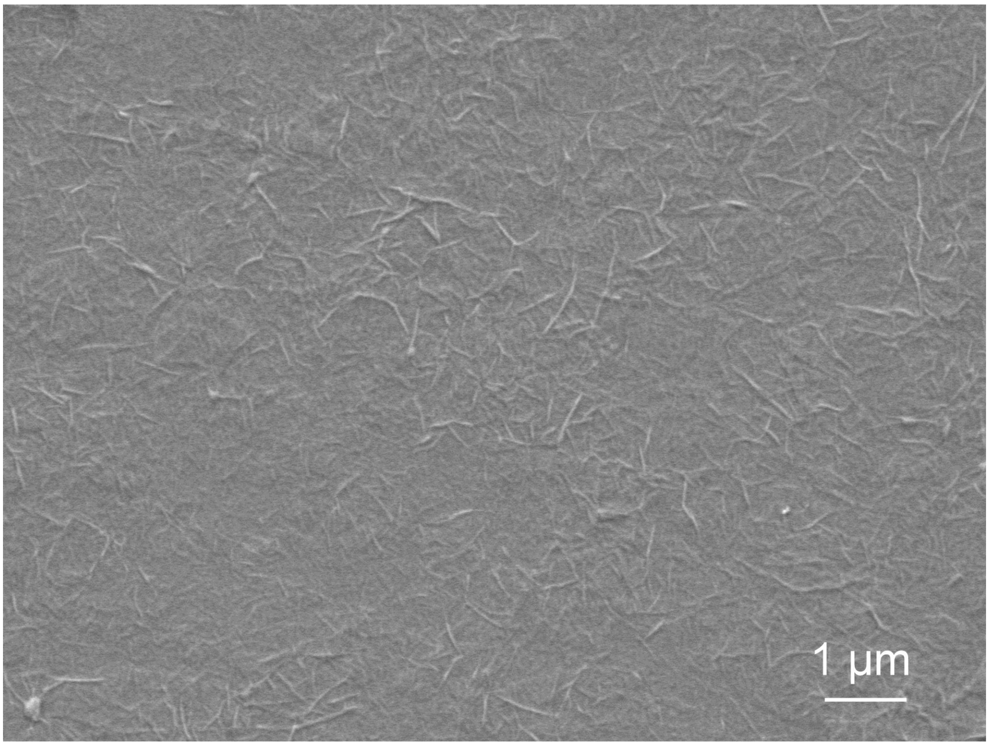

Supplementary Figure 9. Morphology of pure GO film. SEM image of GO transferring to the SiO_2/Si substrate after the toluene solvent evaporates.

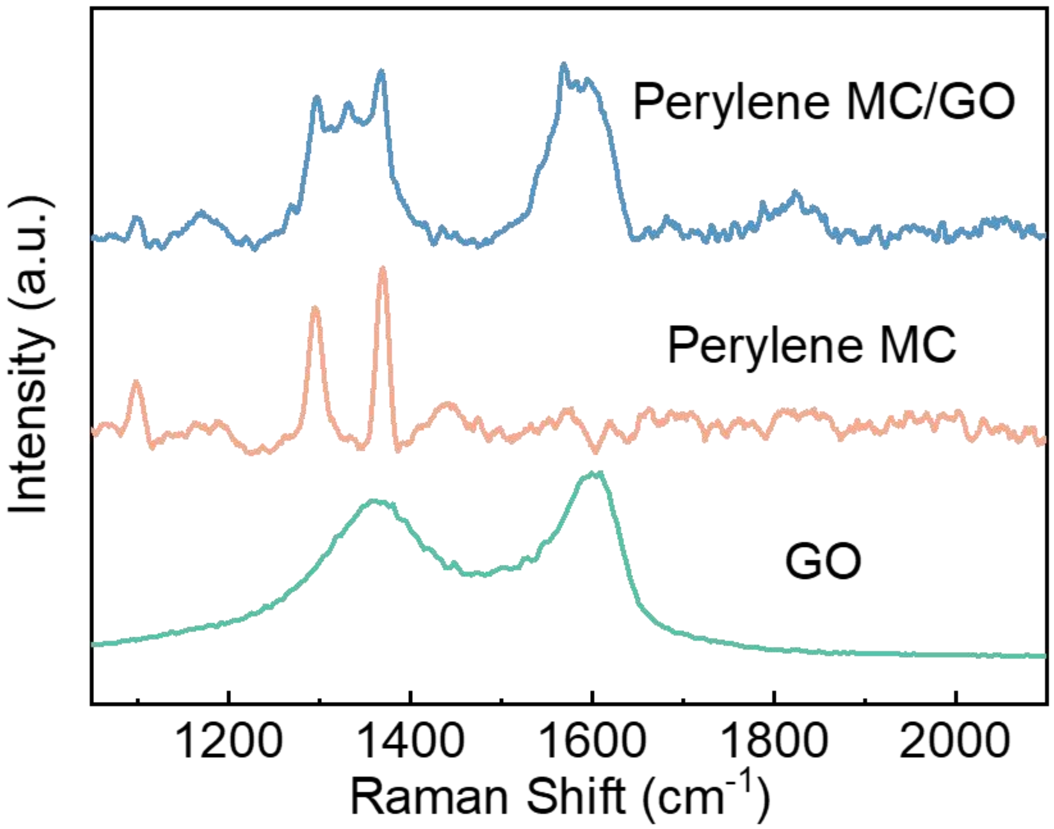

Supplementary Figure 10. Raman spectrum of GO, perylene molecular crystal (MC) and perylene/GO heterobilayer, respectively.

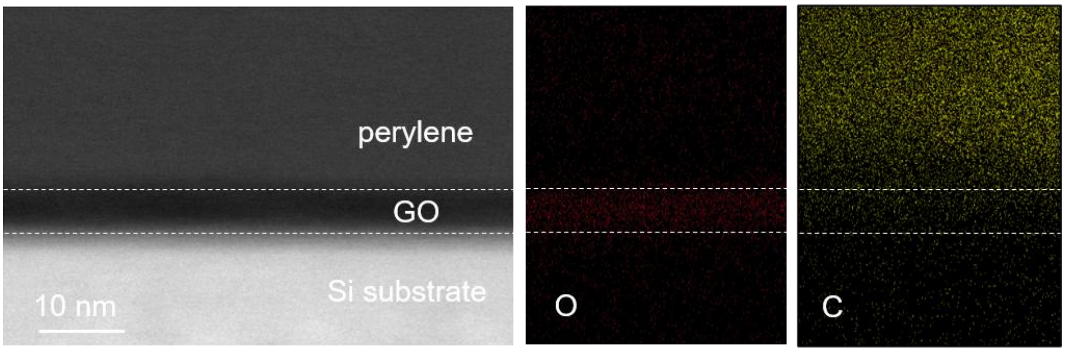

Supplementary Figure 11. Cross-sectional STEM image and corresponding EDS images of perylene/GO heterostructure film transferred onto a Si substrate.

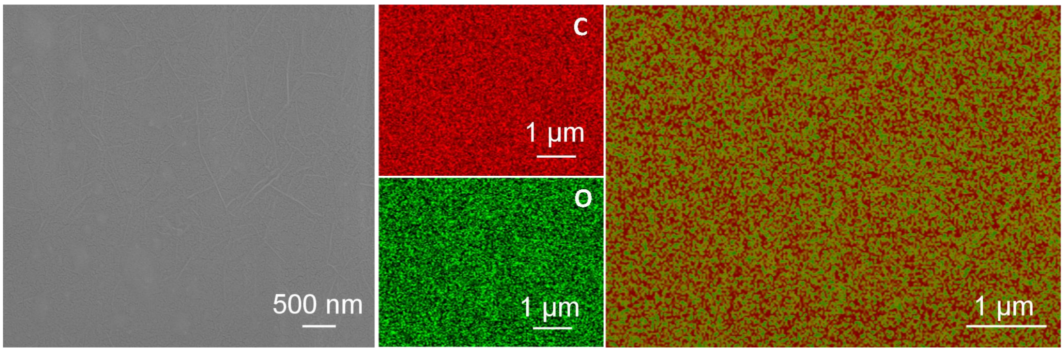

Supplementary Figure 12. SEM images of perylene/GO heterostructure with the upper layer of GO and element mapping of C and O using EDS on SEM.

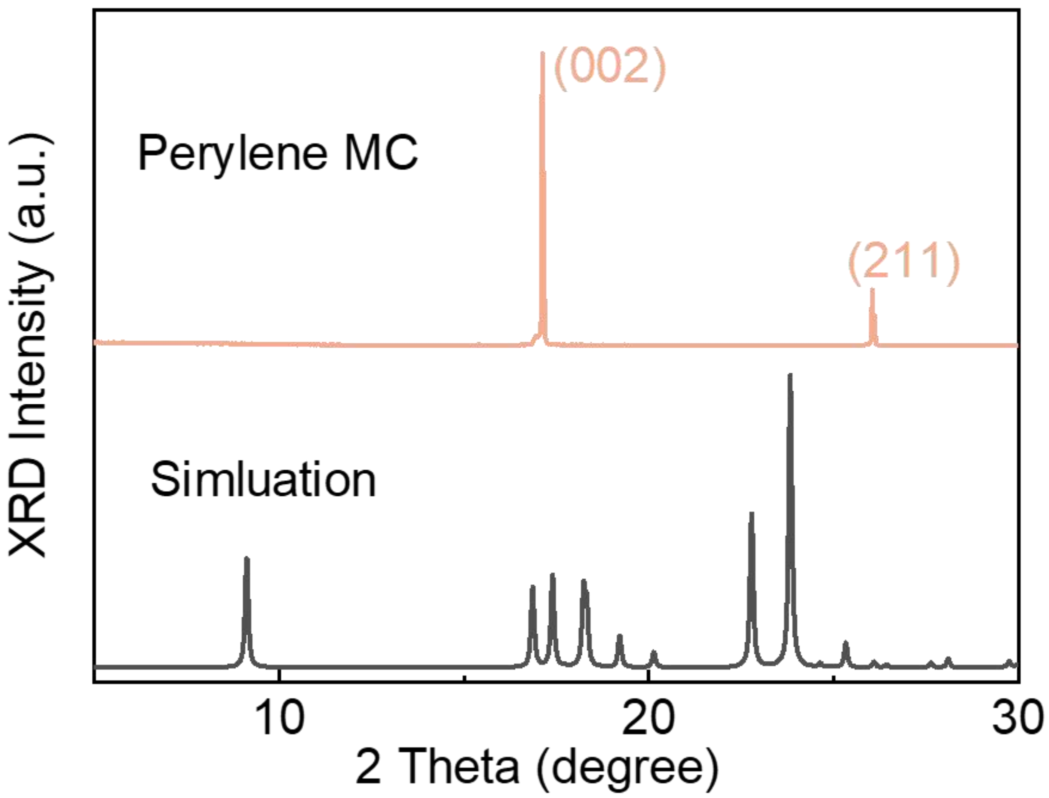

Supplementary Figure 13. XRD patterns of perylene crystal (top) and the simulated XRD based on the single crystal data (bottom). The XRD patterns shows the two-dimensional crystal structure of co-self-assembled perylene, which possesses good photoelectric properties and fast response time.

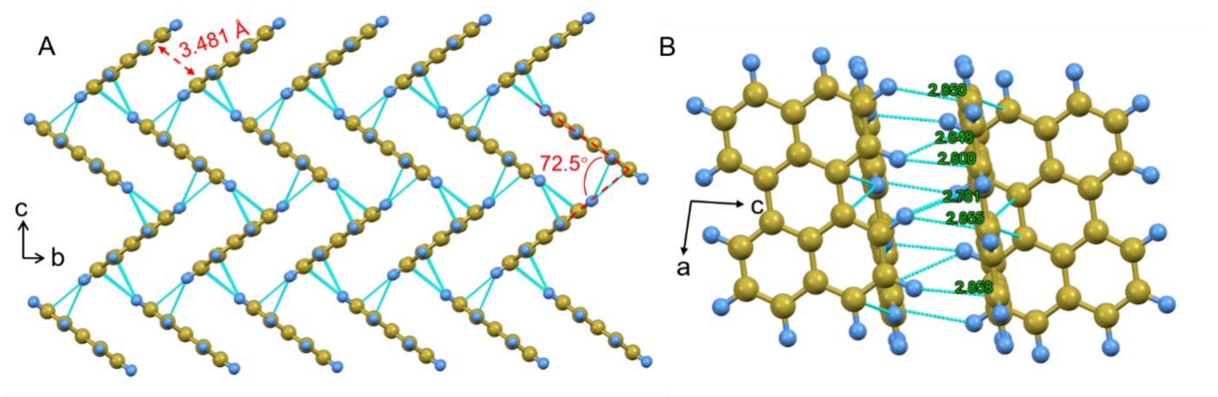

Supplementary Figure 14. Intermolecular interactions in perylene crystal.

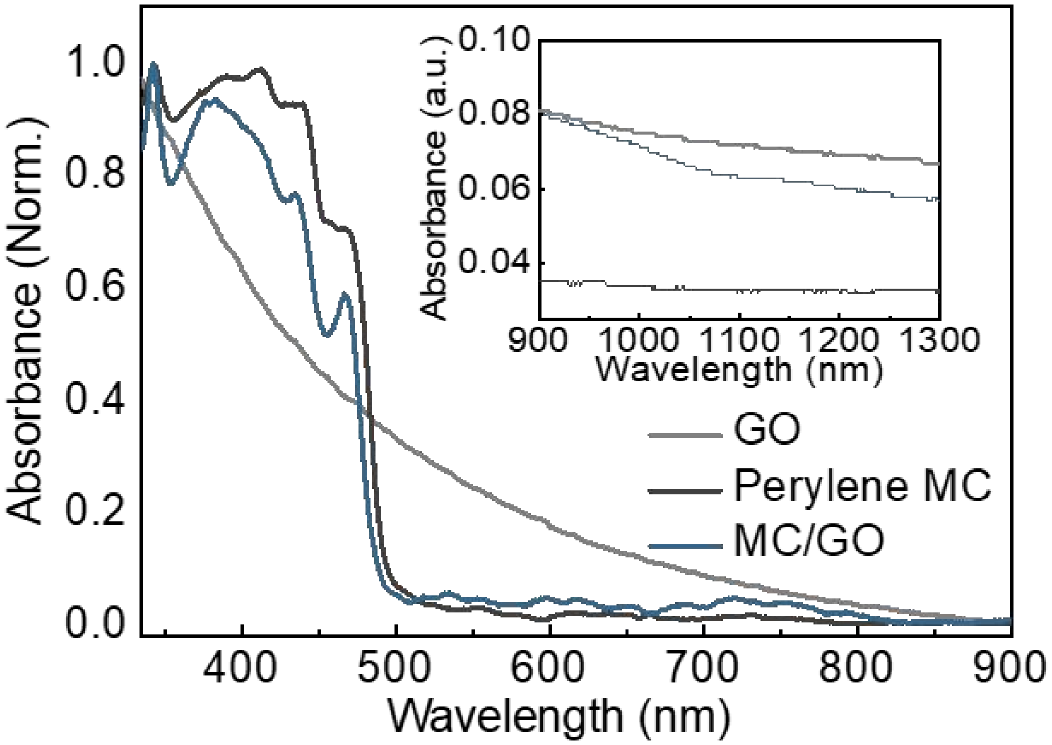

Supplementary Figure 15. UV-visible to NIR absorption spectrum of GO, perylene crystal and perylene/GO heterobilayer. The inset is the NIR absorption spectrum of the three substances.

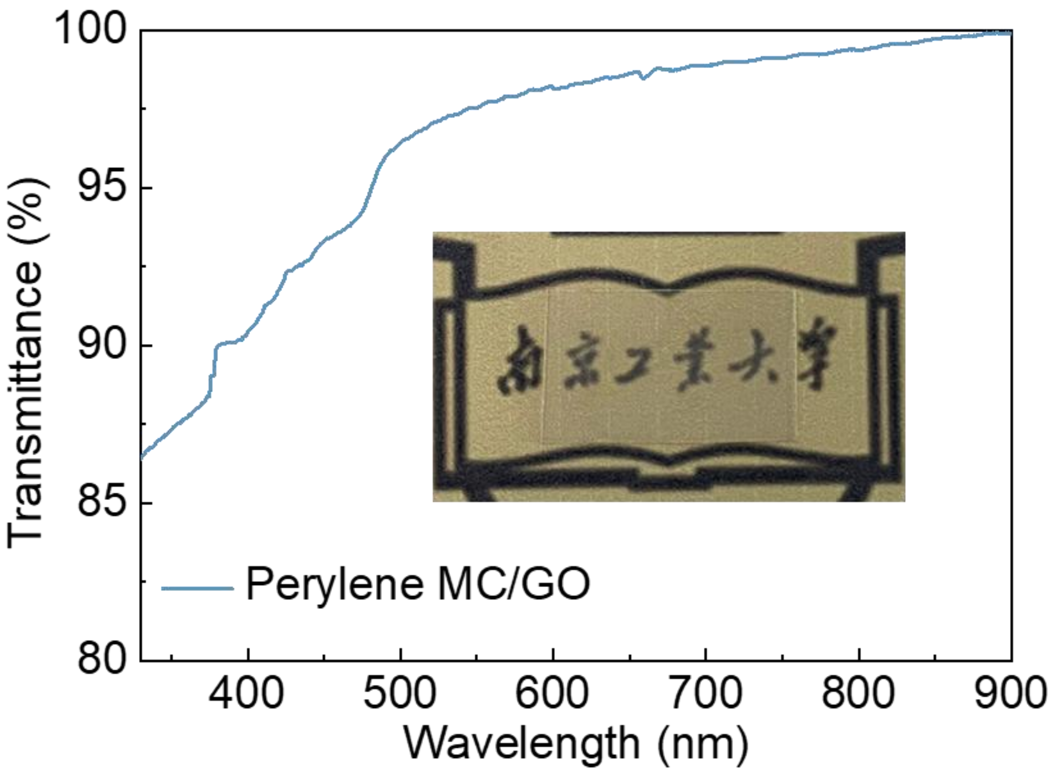

Supplementary Figure 16. Optical transmission spectrum of perylene/GO heterobilayer on quartz substrate. The heterobilayer has a transmittance of about 85% in the visible range, and the pattern Chinese characters below is clearly visible with nearly no color variation.

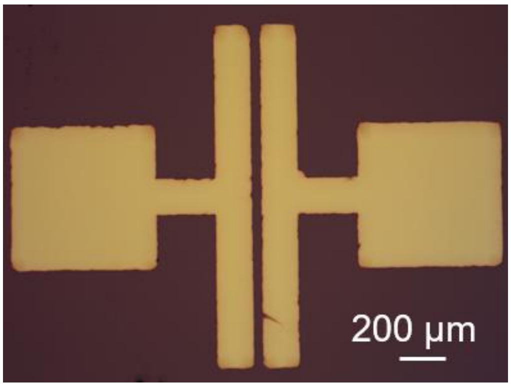

Supplementary Figure 17. OM image of the electrode structure.

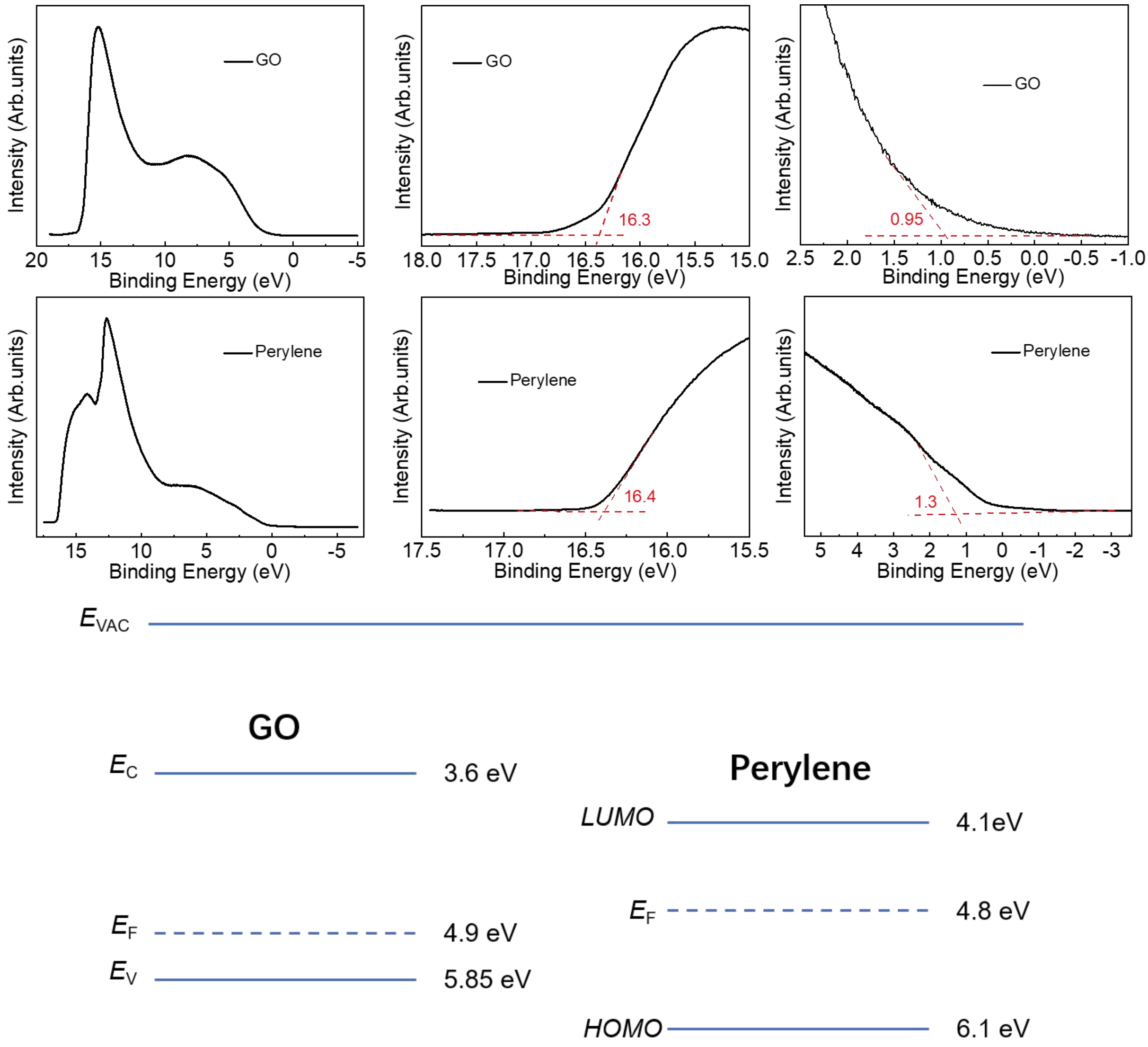

Supplementary Figure 18. Band structure of GO and Perylene. The Fermi energy level positions and valence band positions of GO and perylene are calculated by UV photoelectron spectroscopy (UPS). According to the existing reports[1, 2], the band gap width and conduction band position are deduced, and finally the band structures of the two bands were determined.

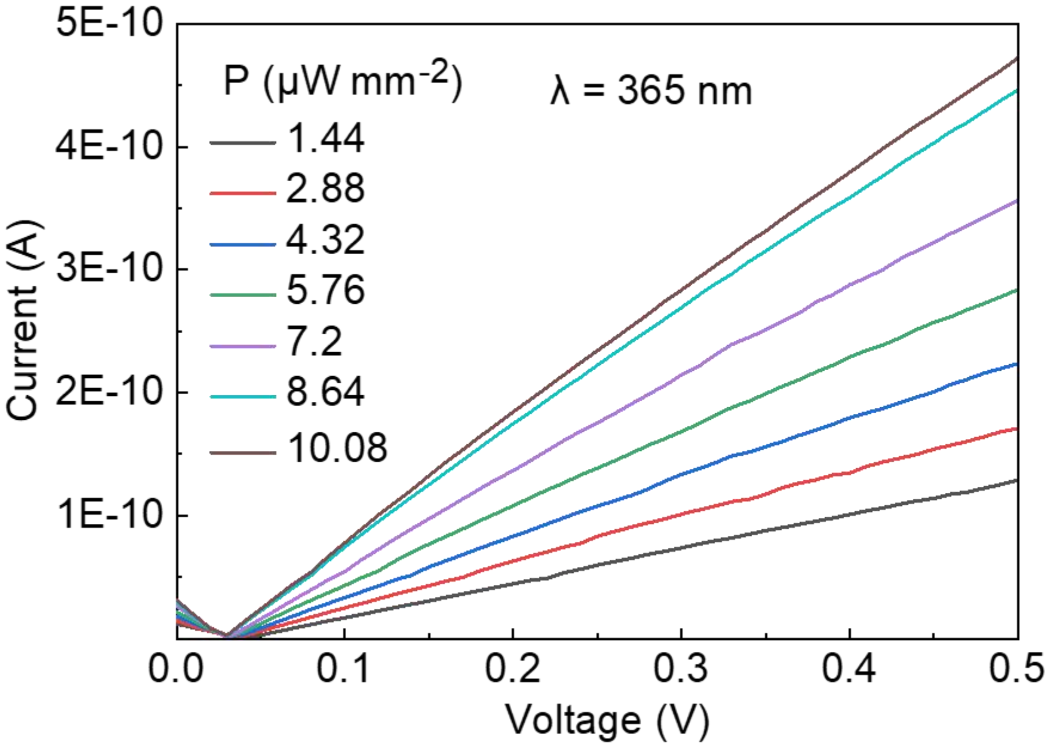

Supplementary Figure 19. I-V characterization curves under different light power at 365 nm. At a sweep voltage of 0 to 0.5 V, a significant current increase occurs as the light intensity increases from 1.44 to 10.08 μW mm^{-2}.

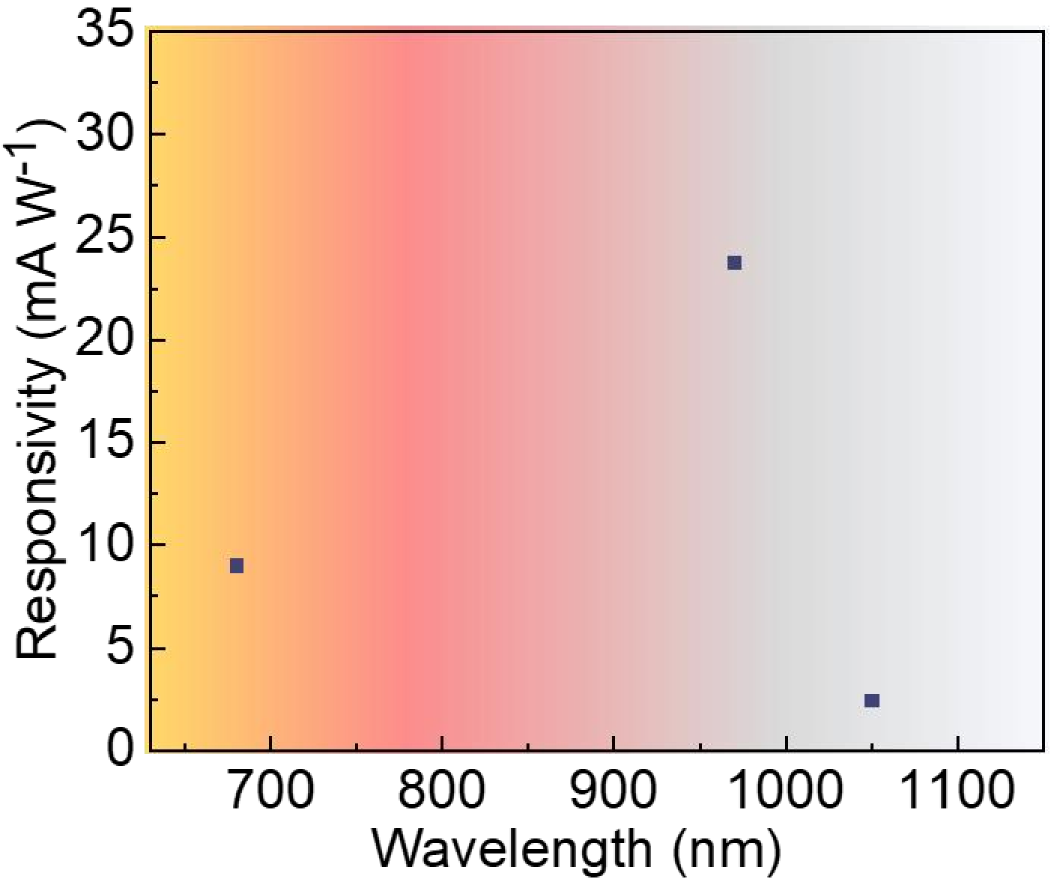

Supplementary Figure 20. Photoresponsivity as a function of light wavelength in the near infrared region. The photoresponsivity of the heterobilayer at 970 nm is even comparable to that of the ultraviolet area due to the absorption of GO material in the infrared region.

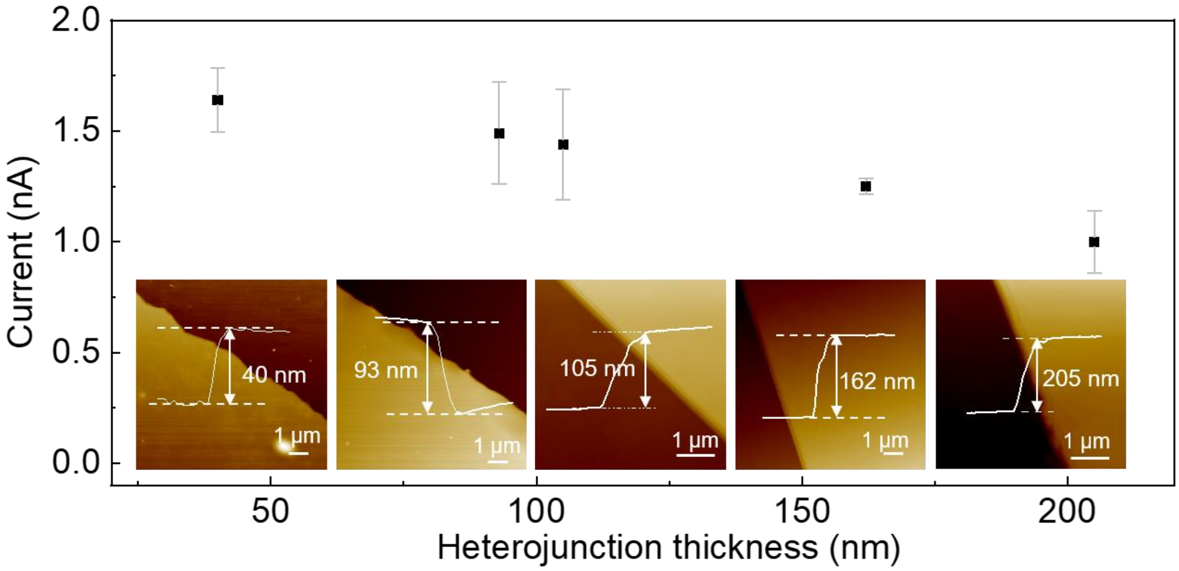

Supplementary Figure 21. The photocurrent of heterojunction with different thickness. The current changes only slightly from 40-200 nm, almost staying around 1 nA.

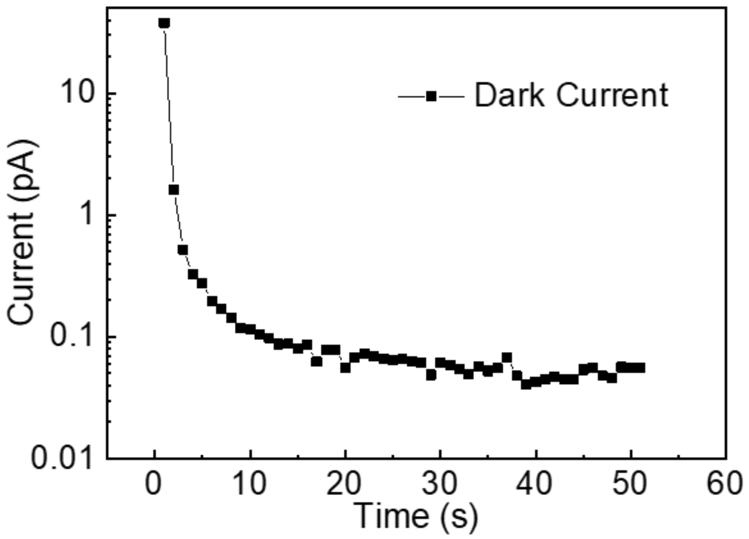

Supplementary Figure 22. Noise current. The current of the device in a dark environment with V_b = 1 V. The current gradually decreases under bias voltage and stabilizes around 0.03 pA, and the ultra-small noise provides excellent light detection performance for the device.

Supplementary Table 1. The performances of heterostructure based photodetectors.

Materials	D* (Jones)	R (A W^{-1})	t	λ (nm)	I_{on}/I_{off}	Voltage (V)	Current (A)	Power (W)	Ref.
Graphene/Perovskite/Graphene	3.4×10^{10}	0.53	/	400-700	10	3	10^{-7}	3×10^{-7}	3
Al Nanostructure/ZnO	/	11.98	0.79 s	365	10^4	10	2.8×10^{-5}	2.8×10^{-4}	4
Graphene/Bi_2Te_3	/	35	8.7 ms	532-1550	1	1	2.5×10^{-5}	2.5×10^{-5}	5
MoS_2/SWCNTs	/	0.1	15 µs	650-1000	10^4	5	4.5×10^{-5}	2.25×10^{-4}	6
Graphene/Silicon	/	0.13	/	1550-2750	30	1.5	6×10^{-7}	9×10^{-7}	7
Graphene/Ta_2O_5/Graphene	/	1	10 ms	532-3200	10	1	3×10^{-5}	3×10^{-5}	8
C8-BTBT/Graphene	/	1.57×10^4	25 ms	355	/	0.1	2.2×10^{-6}	2.2×10^{-7}	9
Bi_2Te_3/Si	2.5×10^{11}	1	100 ms	635	10	5	2×10^{-5}	10^{-4}	10
$PdSe_2$/Perovskite	~10^{13}	0.313	3.5 µs	200-1550	10^4	5	15×10^{-6}	7.5×10^{-5}	11
WSe_2/SnS_2	1.29×10^{13}	244	0.2 s	550-750	10^4	1	10^{-8}	10^{-8}	12
GaSe/GaSb	2.2×10^{12}	0.115	30 µs	400-1800	10^3	1	1.4×10^{-3}	1.4×10^{-3}	13
MoS_2/α-MoO_3-x	1.7×10^{14}	1.9×10^5	95µs	405-638	10^7	1	10^{-5}	10^{-5}	14
ZnO/Ga_2O_3	6.29×10^{12}	9.7×10^{-3}	100 µs	200-280	10^5	2	2×10^{-6}	4×10^{-6}	15
MoS_2/WS_2	/	1.42	/	633	10^3	1	3×10^{-7}	3×10^{-7}	16
MoS_2/Graphene/WSe_2	10^{14}	10^4	53.6 µs	500-2500	10^3	1	1.2×10^{-6}	1.2×10^{-6}	17
GaTe/MoS_2	8.4×10^{13}	21.83	7 ms	473	10^3	1	4.5×10^{-8}	4.5×10^{-8}	18
$PdSe_2$/MoS_2	8.21×10^9	42.1	74.5 ms	10.6 µm	1	1	10^{-5}	10×10^{-6}	19
IGZO/ZnO NRs	8.21×10^{16}	4.9×10^5	/	400	9.5×10^5	60	10^{-6}	6×10^{-5}	20
Chlorophyll/PDPP4T	6×10^{15}	2×10^6	/	/	2.7×10^6	60	10^{-6}	6×10^{-5}	21
C8-BTBT/$CH_3NH_3PbI_3$ NPs	2.09×10^{12}	1.72×10^4	/	252-780	2×10^5	50	2×10^{-5}	10^{-3}	22
This Work	3.1×10^{13}	0.112	50 ms	365-1550	10^3	1	10^{-9}	10^{-9}	

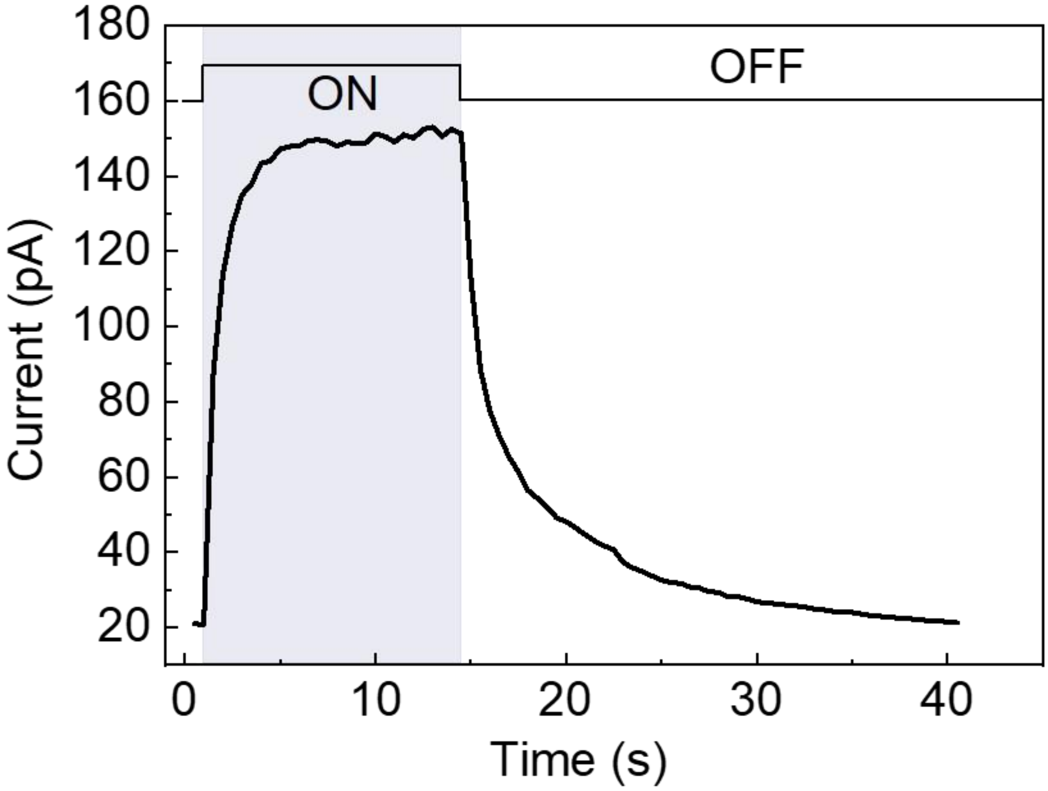

Supplementary Figure 23. Typical photoelectric switching characteristics of the device under 0.144 μW mm^{-2} UV light with V_b = 1 V.

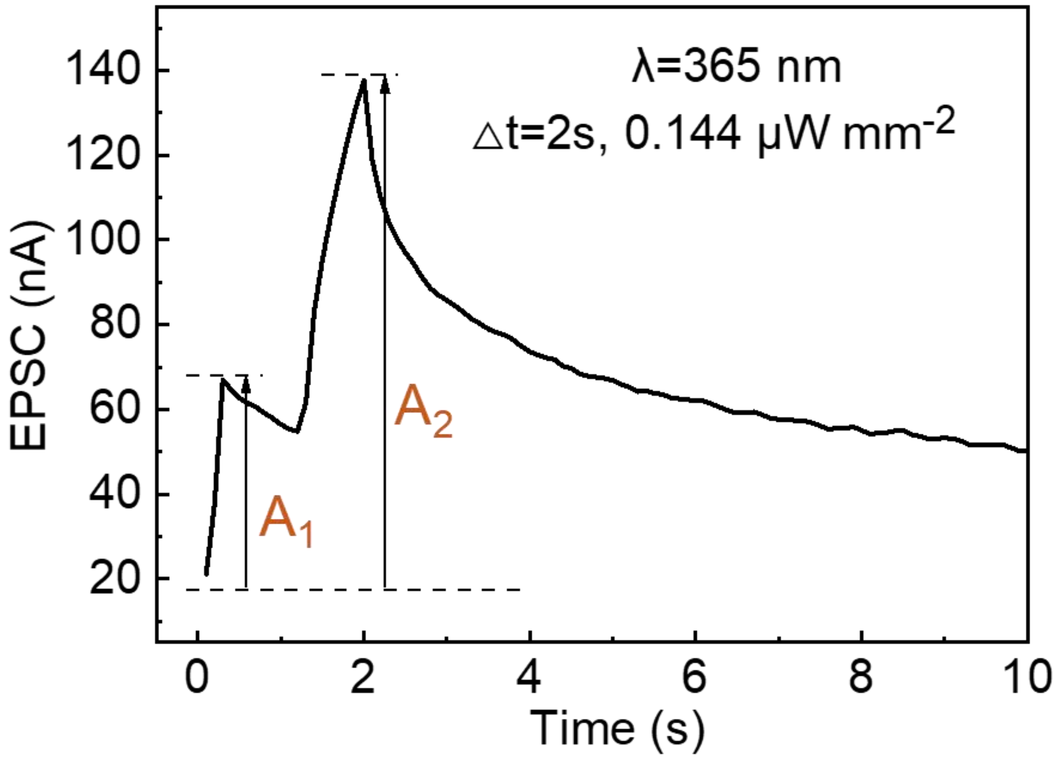

Supplementary Figure 24. Typical paired-pulse facilitation (PPF) behavior of photonic synaptic device (V_b = 1 V). PPF in biological nerve systems refers to an increase in the postsynaptic potential induced by an impulse that occurs immediately after another. PPF is generated by a rise in presynaptic Ca^{2+} concentration, which causes additional synaptic vesicles carrying neurotransmitters to be released. In this device, the ratio of A_2/A_1 is defined as the PPF index.

Supplementary Table 2. Comparison of the maximum PPF index value of artificial synapses.

Device	Active material	PPF index	Ref.
transistor	IGZO/chitosan	137%	23
transistor	PQT_{12}-$CsPbBr_3$ QDs/SiO_2	132%	24
transistor	$CsPbBr_3$/PMMA/pentacene	130%	25
transistor	POx/BP/SiO_2	155%	26
transistor	IGZO/HfZrOx	140%	27
Electrolyte-gated transistor	Indium-zinc oxide thin film	180%	28
Electrolyte-gated transistor	PEO/P3HT core-sheath NWs	162%	29
Electrolyte-gated transistor	Multilayer MoO_3 nanoflake	114%	30
Electrolyte-gated transistor	Indium-tungsten oxide thin film	190%	31
Organic field-effect transistors	P3HT thin film	190%	32
Organic electrochemical transistor	PEDOT:PSS thin film	185%	33
Memristor	Conjugated polyelectrolyte film	145%	34
Memristor	Mesoporous silica	128%	35
Photonic flash memory	Evaporated pentacene thin film	130%	25
Quasi-2DEGs photonic synapses	$InGaO_3(ZnO)_3$ superlattice NWs	210%	36
Two-terminal	ITO/Nb:$SrTiO_3$	144%	37
Two-terminal	Au/LSNO	138%	38
Two-terminal	ZnO1-x/AlOy	170%	39
Two-terminal	In_2O_3/ZnO/FTO	180%	40
This Work	perylene/GO	214%	

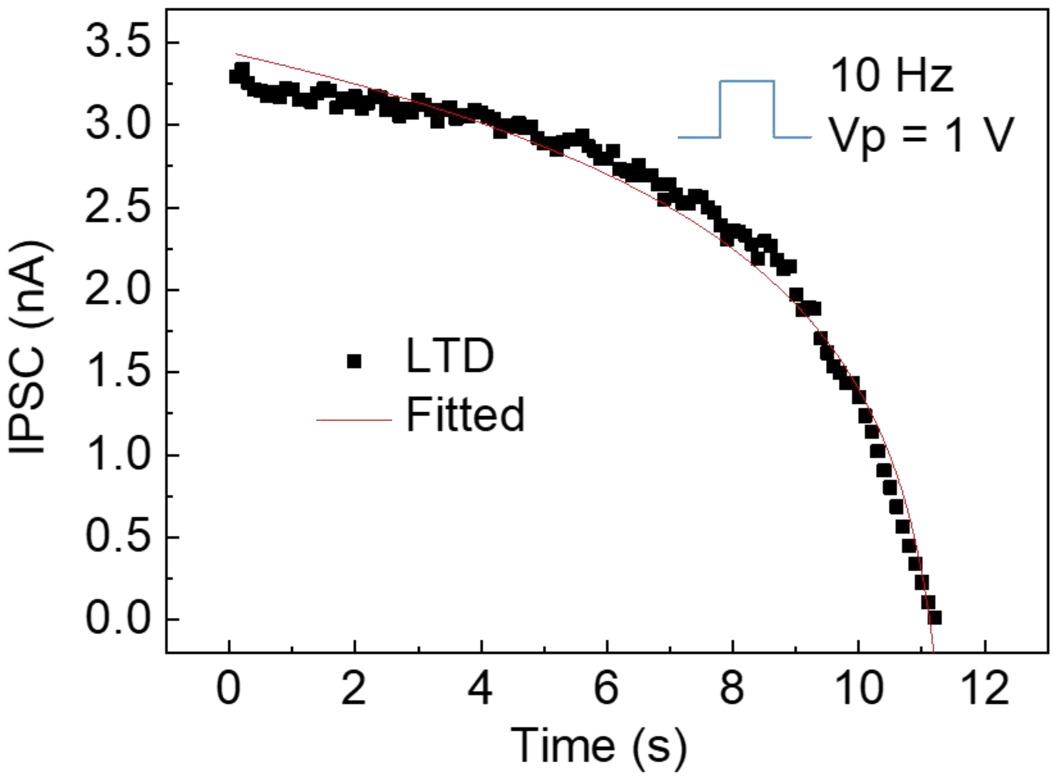

Supplementary Figure 25. Inhibitory postsynaptic potential behavior under pulsed voltage (1 V 10 Hz) after illumination. The device's photocurrent reaches 3.26 nA when exposed to strong light. Pulse voltage is provided to both ends of the device at this time, and the current is suppressed from nA to pA levels.

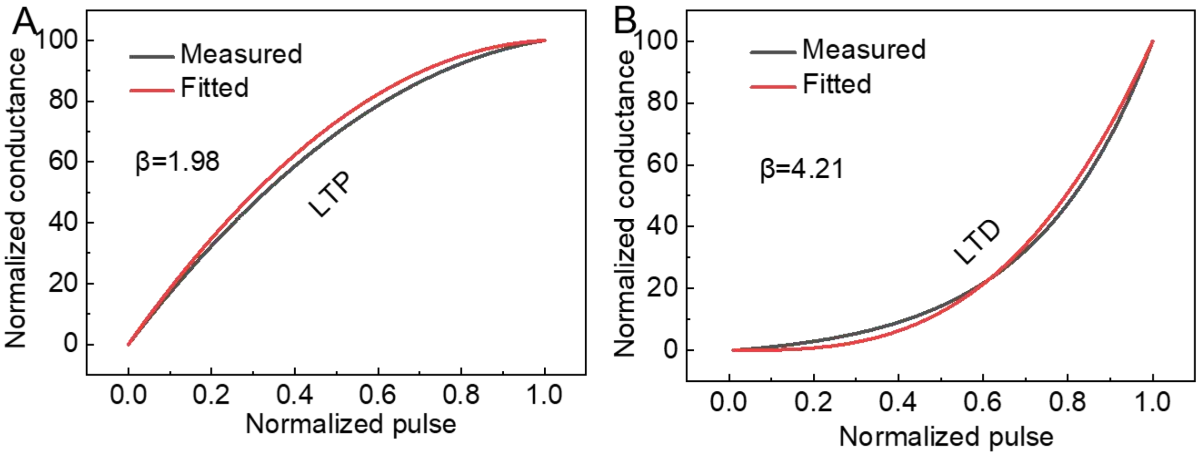

Supplementary Figure 26. Nonlinearity analysis of LTP/LTD curves. A). Fitting curves of b LTP at β_P = 1.98 using the extracted parameters given in Figure 4c. B). Fitting curves of b LTD at β_D = 4.21 using the extracted parameters given in Supplementary Figure 25.

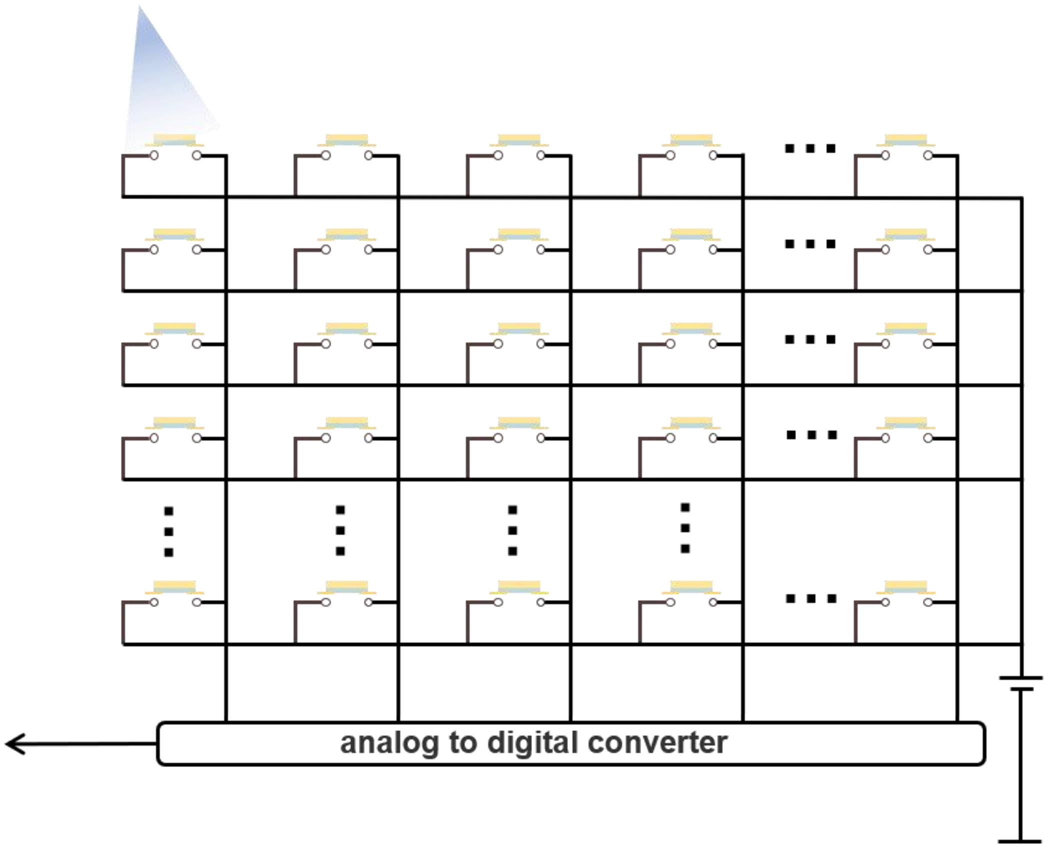

Supplementary Figure 27. Schematic of the hardware implementation of the light-derived neural network by using the photosynapse at each intersection.

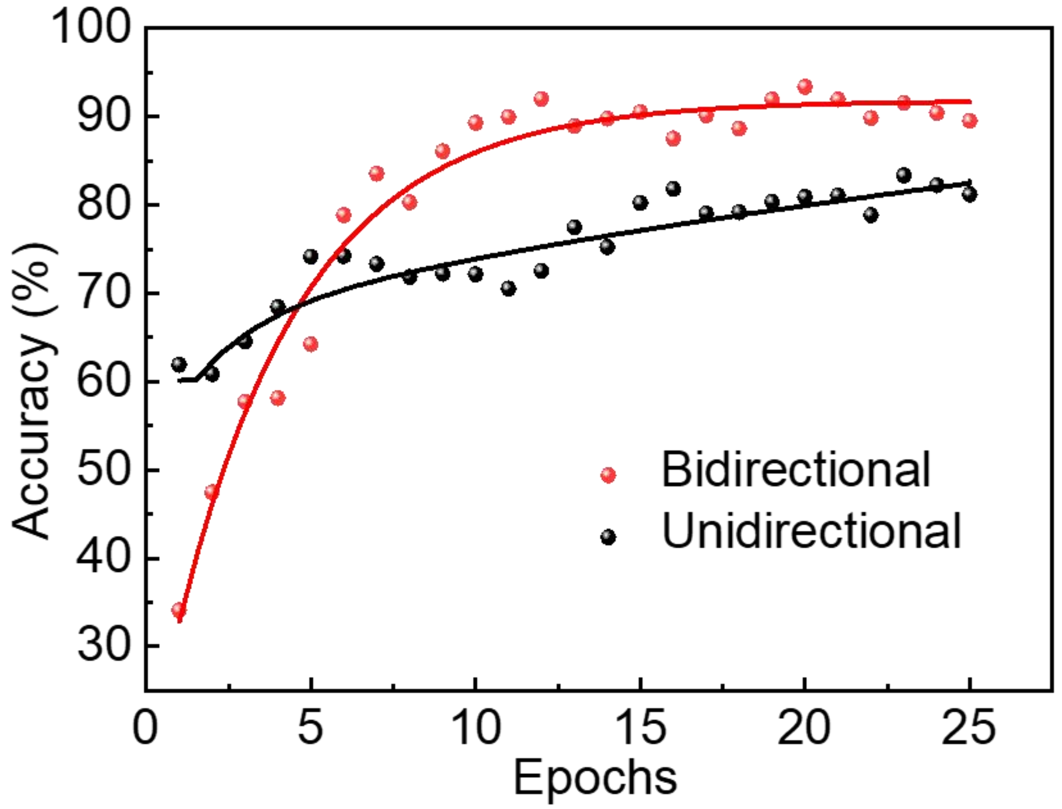

Supplementary Figure 28. Bidirectional and unidirectional number recognition accuracy in the training epochs.

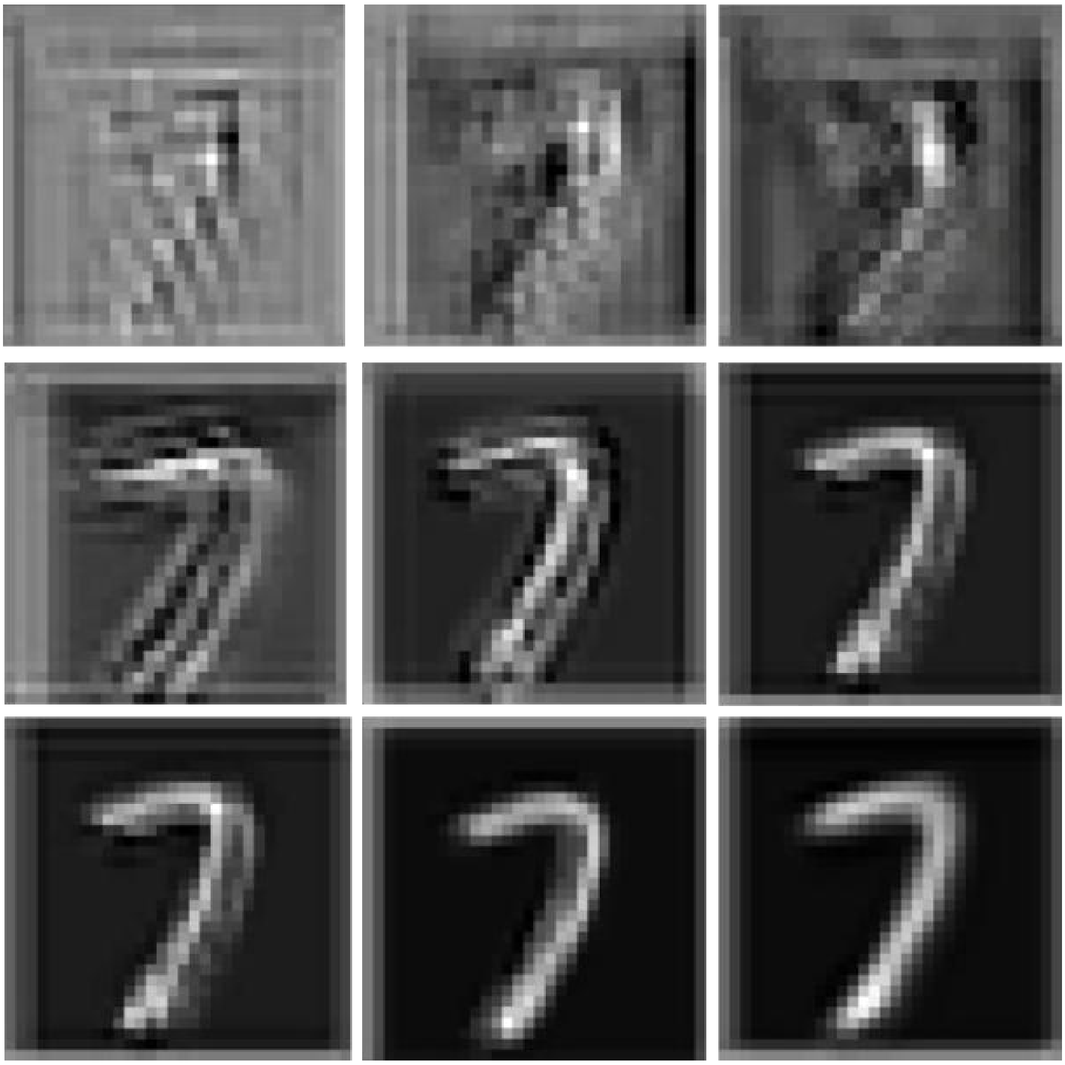

Supplementary Figure 29. Results of a number '7' in the initial state and after training processes.

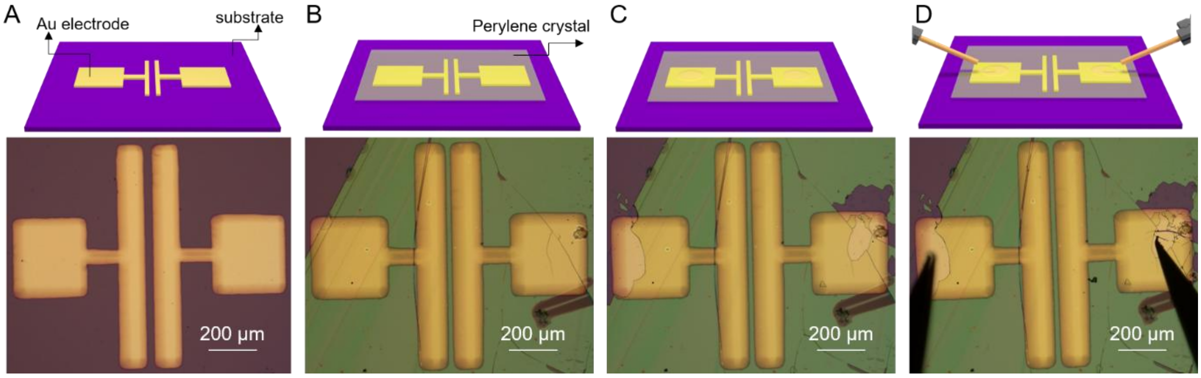

Supplementary Figure 30. Stepwise OM images of device testing. In our work, we use a simple mechanical stripping method, only tape, to remove the upper heterojunction layer without damaging the electrode structure (Figure C). The probe of the semiconductor parameter analyzer can directly contact gold when the pin electrode is totally exposed. The probe is lightly draped over the exposed pin electrodes on either side and the photoelectric properties of the device can be tested without interference from other materials (Figure D).

Supplementary References

1. Pookpanratana, S. J. et al. Electronic properties and structure of single crystal perylene. *Org. Electron.* **61**, 157-163 (2018).
2. Liu, J., Xue, Y., Gao, Y., Yu, D. & Durstock, M. Hole and electron extraction layers based on graphene oxide derivatives for high-performance bulk heterojunction solar cells. *Adv. Mater.* **24**, 2228-2233 (2012).
3. Alamri, A. M., Leung, S., Vaseem, M., Shamim, A. & He, J. H. Fully inkjet-printed photodetector using a graphene/perovskite/graphene heterostructure. *IEEE Trans. Electron. Devices* **66**, 2657-2661 (2019).
4. Liu, S. et al. Self-assembled Al nanostructure/ZnO quantum dot heterostructures for high responsivity and fast UV photodetector. *Nanomicro Lett.* **12**, 114 (2020).
5. Qiao, H. et al. Broadband photodetectors based on graphene–Bi_2Te_3 heterostructure. *ACS Nano* **9**, 1886-1894 (2015).
6. Jariwala, D. et al. Gate-tunable carbon nanotube-MoS_2 heterojunction p-n diode. *Proc. Natl. Acad. Sci. U. S. A.* **110**, 18076-18080 (2013).
7. Wang, X., Cheng, Z., Xu, K., Tsang, H. K. & Xu, J. B. High-responsivity graphene/silicon-heterostructure waveguide photodetectors. *Nat. Photonics* **7**, 888-891 (2013).
8. Liu, C. H., Chang, Y. C., Norris, T. B. & Zhong, Z. Graphene photodetectors with ultra-broadband and high responsivity at room temperature. *Nat. Nanotechnol.* **9**, 273-278 (2014).
9. Liu, X. et al. Epitaxial ultrathin organic crystals on graphene for high-efficiency phototransistors. *Adv. Mater.* **28**, 5200-5205 (2016).
10. Yao, J., Shao, J., Wang, Y., Zhao, Z. & Yang, G. Ultra-broadband and high response of the Bi_2Te_3-Si heterojunction and its application as a photodetector at room temperature in harsh working environments. *Nanoscale* **7**, 12535-12541 (2015).
11. Zeng, L. H. et al. Multilayered $PdSe_2$/perovskite schottky junction for fast, self-powered, polarization-sensitive, broadband photodetectors, and image sensor application. *Adv. Sci.* **6**, 1901134

(2019).
12. Zhou, X. et al. Tunneling diode based on WSe_2/SnS_2 heterostructure incorporating high detectivity and responsivity. *Adv. Mater.* **30**, 1703286 (2018).
13. Wang, P. et al. Arrayed van der Waals broadband detectors for dual-band detection. *Adv. Mater.* **29**, 1604439 (2017).
14. Feng, S. et al. An ultrasensitive molybdenum-based double-heterojunction phototransistor. *Nat. Commun.* **12**, 4094 (2021).
15. Zhao, B. et al. An ultrahigh responsivity (9.7 mA W^{-1}) self-powered solar-blind photodetector based on individual ZnO-Ga_2O_3 heterostructures. *Adv. Funct. Mater.* **27**, 1700264 (2017).
16. Huo, N., Kang, J., Wei, Z., Li, S. S. & Li, J. Novel and enhanced optoelectronic performances of multilayer MoS_2-WS_2 heterostructure transistors. *Adv. Funct. Mater.* **24**, 7025-7031 (2014).
17. Long, M. et al. Broadband photovoltaic detectors based on an atomically thin heterostructure. *Nano Lett.* **16**, 2254-2259 (2016).
18. Wang, F. et al. Tunable GaTe-MoS_2 van der Waals p-n junctions with novel optoelectronic performance. *Nano Lett.* **15**, 7558-7566 (2015).
19. Long, M. et al. Palladium diselenide long-wavelength infrared photodetector with high sensitivity and stability. *ACS Nano* **13**, 2511-2519 (2019).
20. Tao, Z., Liu, X., Lei, W. & Chen, J. High sensitive solar blind phototransistor based on ZnO nanorods/IGZO heterostructure annealed by laser. *Mater. Lett.* **228**, 451-455 (2018).
21. Yang, B. et al. Bioinspired multifunctional organic transistors based on natural chlorophyll/organic semiconductors. *Adv. Mater.* **32**, e2001227 (2020).
22. Xu, X. et al. Dual-band, high-performance phototransistors from hybrid perovskite and organic crystal array for secure communication applications. *ACS Nano* **13**, 5910-5919 (2019).
23. Yang, Y., He Y., Nie, S., Shi, Y. & Wan, Q. Light stimulated IGZO-based electric-double-layer transistors for photoelectric neuromorphic devices. *IEEE Electron. Device Lett.* **39**, 897-900 (2018)
24. Wang, K., Dai, S., Zhao, Y., Wang, Y., Liu, C. & Huang, J. Light-stimulated synaptic transistors fabricated by a facile solution process based on inorganic perovskite quantum dots and organic semiconductors. *Small* **15**, 1900010 (2019).
25. Wang, Y. et al. Photonic synapses based on inorganic perovskite quantum dots for neuromorphic computing. *Adv. Mater.* **30**, 1802883 (2018).
26. Tian, H. et al. Anisotropic black phosphorus synaptic device for neuromorphic applications. *Adv. Mater.* **28**, 4991-4997 (2016).
27. Kim, M. K. & Lee, J. S. Synergistic improvement of long-term plasticity in photonic synapses using ferroelectric polarization in hafnia-based oxide-semiconductor transistors. *Adv. Mater.* **32**, e1907826 (2020).
28. Zhu, L. Q., Wan, C. J., Guo, L. Q., Shi, Y. & Wan, Q. Artificial synapse network on inorganic proton conductor for neuromorphic systems. *Nat. Commun.* **5**, 3158 (2014).
29. Xu, W., Min, S. Y., Hwang, H. & Lee, T. W. Organic core-sheath nanowire artificial synapses with femtojoule energy consumption. *Sci. Adv.* **2**, e1501326 (2016).
30. Yang, C.S. et al. A synaptic transistor based on quasi-2D molybdenum oxide. *Adv. Mater.* **29**, 1700906 (2017).
31. Wan, C. et al. An artificial sensory neuron with tactile perceptual learning. *Adv. Mater.* **30**, 1801291 (2018).
32. Zang. Y., Shen, H., Huang, D., Di, C. A. & Zhu, D. A dual-organic-transistor-based tactile-perception

system with signal-processing functionality. *Adv. Mater.* **29**, 1606088 (2017).

33. Gkoupidenis, P., Schaefer, N., Garlan, B. & Malliaras, G. G. Neuromorphic functions in PEDOT: PSS organic electrochemical transistors. *Adv. Mater.* **27**, 7176-7180 (2015).
34. Xu, W. et al. Ultrasensitive artificial synapse based on conjugated polyelectrolyte. *Nano Energy* **48**, 575-581 (2018).
35. Li, B. et al. Mediating short-term plasticity in an Artificial memristive synapse by the orientation of silica mesopores. *Adv. Mater.* **30**, 1706395 (2018).
36. Meng, Y. et al. Artificial visual systems enabled by quasi-two-dimensional electron gases in oxide superlattice nanowires. *Sci. Adv.* **6**, eabc6389 (2020).
37. Gao, S. et al. An oxide Schottky junction artificial optoelectronic synapse. *ACS Nano* **13**, 2634-2642 (2019).
38. Zhao, L. et al. An artificial optoelectronic synapse based on a photoelectric memcapacitor. *Adv. Electron. Mater.* **6**, 1900858 (2020).
39. Hu, D. C., Yang, R., Jiang, L. & Guo, X. Memristive synapses with photoelectric plasticity realized in ZnO_{1-x}/AlO_y heterojunction. *ACS Appl. Mater. Interfaces* **10**, 6463-6470 (2018).
40. Kumar, M., Abbas. S. & Kim, J. All-oxide-based highly transparent photonic synapse for neuromorphic computing. *ACS Appl. Mater. Interfaces* **10**, 34370-34376 (2018).
